# TCF7L2 promotes abdominal aortic aneurysm through smooth muscle cell–mediated extracellular matrix remodeling

**DOI:** 10.1172/jci.insight.195681

**Published:** 2026-04-30

**Authors:** Yongjie Deng, Yaozhong Liu, Yang Zhao, Hongyu Liu, Guizhen Zhao, Zhenguo Wang, Xu Zhang, Chao Xue, Wei Huang, Tianqing Zhu, Haocheng Lu, Yanhong Guo, Lin Chang, Ida Surakka, Y. Eugene Chen, Jifeng Zhang

**Affiliations:** 1 Department of Internal Medicine, Frankel Cardiovascular Center, University of Michigan Medical Center, Ann Arbor, Michigan, USA.; 2 Department of Cardiovascular Medicine, The Second Xiangya Hospital of Central South University, Changsha, China.; 3 Department of Cardiology, Shanghai Sixth People’s Hospital Affiliated to Shanghai Jiao Tong University School of Medicine, Shanghai, China.; 4 Department of Pharmacological and Pharmaceutical Sciences, University of Houston College of Pharmacy, Houston, Texas, USA.; 5 Department of Internal Medicine, Rochester General Hospital, Rochester, New York, USA.; 6 Department of Pharmacology, School of Medicine, Southern University of Science and Technology, Shenzhen, China.

**Keywords:** Cardiology, Cell biology, Vascular biology, Cardiovascular disease, Extracellular matrix

## Abstract

Abdominal aortic aneurysm (AAA) lacks effective pharmacological therapies. Here, we investigate transcription factor 7–like 2 (TCF7L2), a genetic locus associated with both thoracic and abdominal aortic aneurysms, to elucidate its role in AAA pathogenesis. Integrating summary data–based Mendelian randomization (SMR) with single-cell RNA sequencing of human and mouse aortae, we identify *TCF7L2* as a gene enriched in vascular smooth muscle cells (VSMCs) and causally linked to AAA development. Smooth muscle cell–specific TCF7L2 knockout significantly attenuates AAA formation across 3 distinct murine models (AAA induced by angiotensin II infusion, by β-aminopropionitrile/angiotensin II coadministration, and by elastase), independent of systemic blood pressure or lipid levels. Mechanistic studies reveal that TCF7L2 directly upregulates MMP14 and downregulates TIMP3 expression in vitro and in vivo, driving MMP2-mediated extracellular matrix (ECM) degradation. Concurrently, TCF7L2 represses integrin β_1_ (ITGB1) expression, reducing VSMC adhesion to the ECM. Collectively, these findings identify TCF7L2 as a key driver of pathological vascular remodeling in AAA, suggesting that targeting TCF7L2 may offer a novel therapeutic strategy for limiting AAA progression.

## Abstract 2

TCF7L2 is a key driver of pathological remodeling in abdominal aortic aneurysm.

## Graphical abstract

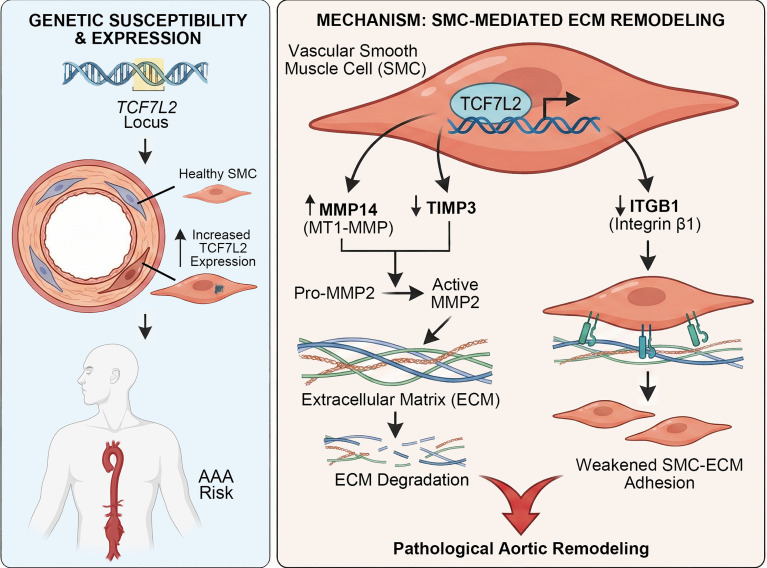

## Introduction

Abdominal aortic aneurysm (AAA) is a life-threatening vascular disorder characterized by progressive dilation of the abdominal aorta, ultimately leading to a high risk of rupture and significant mortality (1). Risk factors such as advanced age, male sex, family history, smoking, hypertension, hyperlipidemia, and history of heart disease contribute to AAA development (2–6). The underlying pathology is driven by vascular smooth muscle cell (VSMC) loss, extracellular matrix (ECM) degradation, and chronic inflammation, which collectively weaken the aortic wall (7, 8). VSMCs play a central role in AAA development through phenotypic switching, apoptosis, metabolic alterations, and dysregulated ECM remodeling (9). Despite extensive research, no pharmacological therapies have been proven to effectively slow AAA progression, leaving surgical repair as the only treatment (1). This highlights the urgent need to elucidate the molecular mechanisms governing AAA pathogenesis to identify novel therapeutic targets.

Genome-wide association studies (GWAS) have substantially advanced our understanding of genetic contributions to complex diseases by identifying susceptibility loci, many of which reside in non-coding regions and likely exert their effects by altering gene expression (10). Our recent large-scale meta-analysis identified 141 independent loci associated with AAA risk (11). Notably, among these genetic loci, transcription factor 7–like 2 (TCF7L2) has emerged as a shared genetic risk factor for both AAA and thoracic aortic aneurysm (11, 12), despite their distinct clinical and pathological features (13). Originally identified as a major risk locus for type 2 diabetes (14), *TCF7L2* encodes a transcription factor central to the canonical Wnt/β-catenin signaling pathway, governing cellular processes such as proliferation, differentiation, apoptosis, and metabolism (15). Together, these findings suggest that TCF7L2 may contribute to aneurysm susceptibility; however, its functional contribution to AAA and the underlying mechanisms remain largely unexplored.

In this study, we identify TCF7L2 as a critical transcriptional regulator in VSMCs that drives AAA development. Using summary data–based Mendelian randomization (SMR), we establish a causal link between TCF7L2 expression and AAA susceptibility. Through integrated in vivo and in vitro studies, we demonstrate that TCF7L2 promotes AAA progression primarily by enhancing ECM remodeling — upregulating MMP14, suppressing TIMP3, and thereby increasing MMP2 activation and ECM degradation. TCF7L2 also reduces VSMC-ECM adhesion by transcriptionally repressing *ITGB1*. Taken together, these changes in ECM turnover and VSMC-matrix adhesion establish TCF7L2 as a key regulator of AAA pathogenesis and highlight it as a potential therapeutic target.

## Results

### Causal link between TCF7L2 expression and elevated risk of AAA.

**Figure 1:**
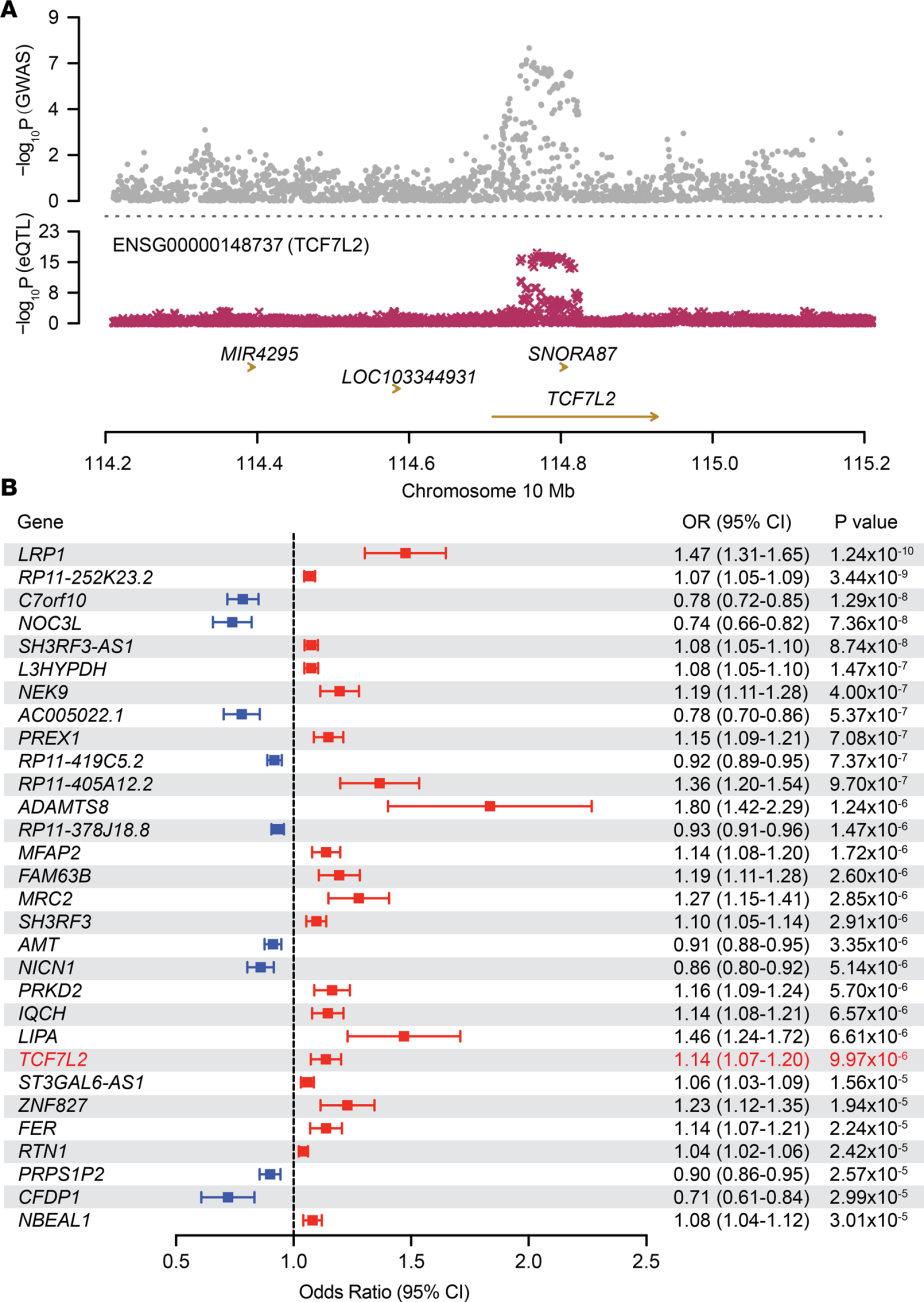
SMR identifies *TCF7L2* as a putative causal gene for AAA susceptibility. (**A**) Locus plot displaying the colocalization of GWAS and eQTL signals at the *TCF7L2* locus on chromosome 10. The top panel shows –log_10_(*P*) values for single-nucleotide polymorphisms (SNPs) from the AAA GWAS meta-analysis (*n* = 39,221 cases and 1,086,107 controls), and the bottom panel shows the –log_10_(*P*) values for SNPs associated with TCF7L2 expression in the human aorta (GTEx v8). Genomic position is indicated on the *x* axis (hg19). (**B**) Forest plot of the top 30 genes prioritized by SMR analysis based on their inferred causal association with AAA. Genes are ranked by increasing *P*_SMR_ and filtered by HEIDI test (*P*_HEIDI_ > 0.05). Odds ratios (ORs) and 95% confidence intervals (CIs) are shown. Red boxes indicate genes whose higher expression is associated with increased AAA risk (OR > 1); blue boxes indicate protective associations (OR < 1).

To investigate whether TCF7L2 expression in the aorta contributes causally to AAA risk, we performed SMR analysis by integrating AAA GWAS data (11) and aortic expression quantitative trait loci (eQTL) information (GTEx v8). The AAA risk variants within the *TCF7L2* locus on chromosome 10 strongly colocalized with the *cis*-eQTL of aortic *TCF7L2* expression (Figure 1A). Notably, the sentinel variant rs6585200 emerged as both a significant AAA-associated single-nucleotide polymorphism and a leading *cis*-eQTL for *TCF7L2*, suggesting that this variant may influence AAA risk by modulating *TCF7L2* expression.

Consistent with this hypothesis, the SMR analysis revealed that a 1-standard-deviation increase in aortic *TCF7L2* expression was associated with a 13% elevated risk of AAA (OR = 1.13, 95% CI: 1.07–1.20, *P* < 1 × 10^–5^), and this relationship remained significant after Benjamini-Hochberg false discovery rate (FDR) correction (*q* = 0.0025). Further evaluation using the heterogeneity in dependent instruments (HEIDI) test showed no evidence of heterogeneity (*P*_HEIDI_ = 0.38), ruling out potential confounding by linkage disequilibrium with neighboring loci (Figure 1B). These findings support a causal contribution of aortic TCF7L2 expression to AAA risk rather than a secondary correlation.

### TCF7L2 exhibits high arterial expression and is predominantly enriched in VSMCs.

To determine the tissue- and cell-specific expression of TCF7L2, particularly within the vasculature, we analyzed bulk RNA sequencing (RNA-seq) data from the Genotype-Tissue Expression (GTEx) project. *TCF7L2* exhibited broad expression across human tissues, with notably higher levels in the aorta and other arterial tissues (Supplemental Figure 1A; supplemental material available online with this article; https://doi.org/10.1172/jci.insight.195681DS1), suggesting a potential role in vascular biology. Complementary analysis of our human abdominal aortic RNA-seq dataset, derived from non-aneurysmal donor aortae (16), further revealed that TCF7L2 and TCF7L1 were the predominantly expressed members of the TCF/LEF transcription factor family, whereas TCF7 and LEF1 showed lower abundance (Supplemental Figure 1B).

We next examined the primary cellular distribution of *TCF7L2* within the aorta using single-cell RNA-seq datasets from both non-aneurysmal human (Gene Expression Omnibus [GEO] GSE155468) and non-aneurysmal mouse (GSE152583) aortic tissues. In the human dataset, uniform manifold approximation and projection (UMAP) clustering identified major aortic cell populations, including VSMCs, fibroblasts, endothelial cells, macrophages, and immune cells. *TCF7L2* expression was predominantly enriched in the VSMC cluster, exhibiting a pattern consistent with canonical VSMC markers such as *MYH11*, *ACTA2*, and *TAGLN* (Supplemental Figure 1, C and D). A similar VSMC-enriched expression pattern was observed in the mouse dataset, indicating conservation across species (Supplemental Figure 1, E and F).

Collectively, these findings demonstrate that *TCF7L2* is preferentially expressed in VSMCs within the aortic wall, establishing a baseline expression pattern that supports its potential involvement in AAA pathogenesis through modulation of VSMC biology.

### Smooth muscle–specific TCF7L2 deficiency attenuates angiotensin II–induced AAA formation.

To directly examine the role of VSMC-derived TCF7L2 in AAA development, we generated mice with smooth muscle cell–specific *Tcf7l2* knockout (*Tcf7l2^SMKO^*) by crossing *Tcf7l2^fl/fl^* mice with *Myh11*-CreER^T2^ transgenic mice (Supplemental Figure 2A). PCR-based genotyping confirmed successful Cre-mediated recombination, yielding *Tcf7l2^SMKO^* mice (Supplemental Figure 2B). Following tamoxifen administration (75 mg/kg/d for 5 consecutive days), efficient *Tcf7l2* deletion in the aortic tissues of *Tcf7l2^SMKO^* mice was validated by Western blot 2 weeks after the first dose (Supplemental Figure 2C).

**Figure 2:**
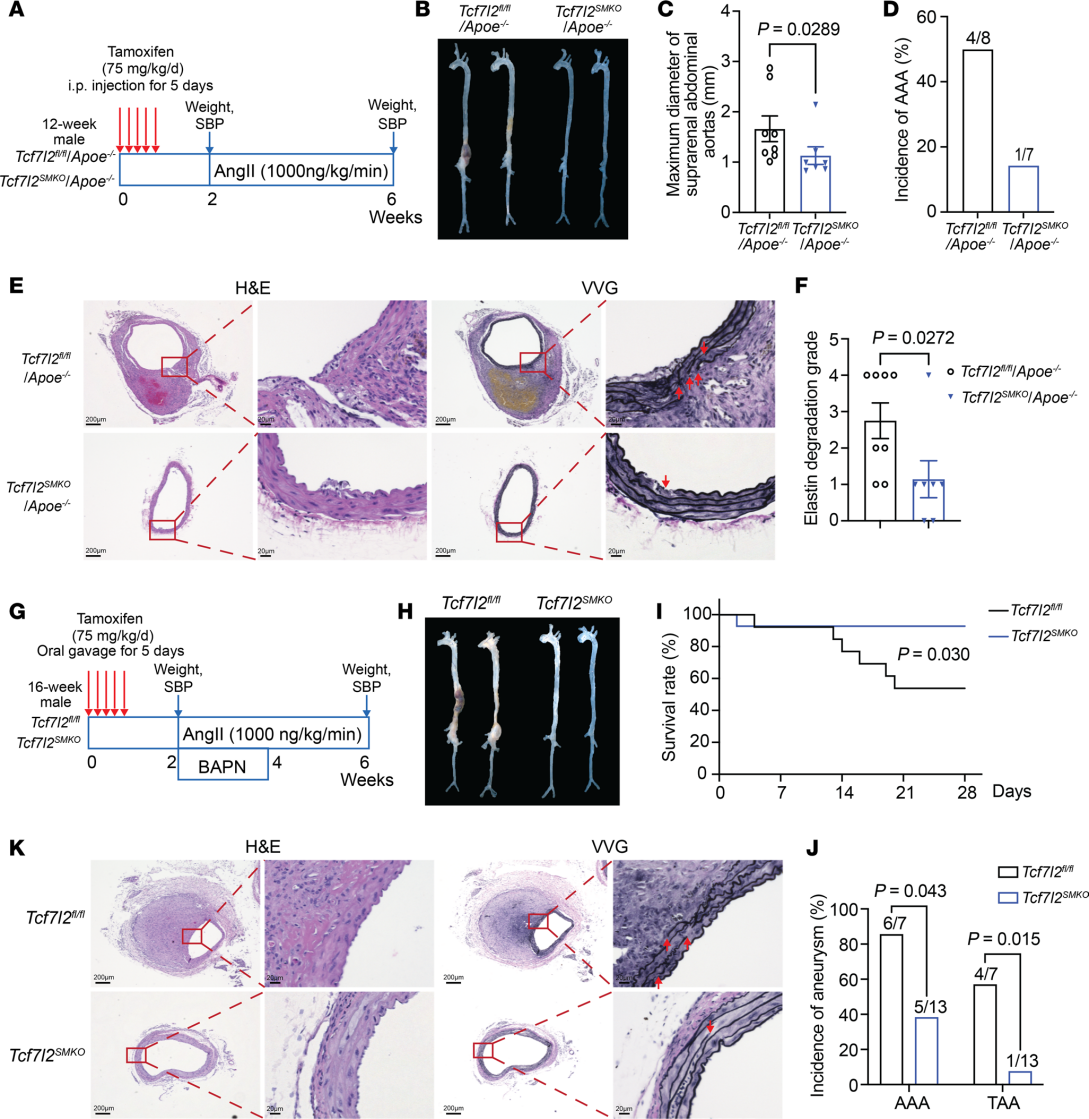
VSMC-TCF7L2 deficiency attenuates Ang II and BAPN/Ang II–induced AAA in mice. (**A**–**F**) In the angiotensin II–induced (Ang II–induced) AAA model, 12-week-old male *Tcf7l2^fl/fl^* *Apoe^–/–^* (*n* = 8) and *Tcf7l2^SMKO^* *Apoe^–/–^* (*n* = 7) mice were infused with Ang II (1,000 ng/kg/min) for 4 weeks. (**A**) Schematic of Ang II–induced AAA model. (**B**) Representative morphology of aortae at the endpoint. (**C**) Maximum diameter of the suprarenal abdominal aortae. (**D**) Incidence of AAA formation. (**E**) Representative images of H&E and VVG staining of suprarenal abdominal aortae; red arrows indicate elastin degradation. Scale bars: 200 μm; 20 μm (higher-magnification images). (**F**) Grade of elastin degradation. (**G**–**K**) In the BAPN/Ang II–induced AAA model, 16-week-old male *Tcf7l2^fl/fl^* (*n* = 13) and *Tcf7l2^SMKO^* (*n* = 14) mice were infused with both Ang II (1,000 ng/kg/min, 4 weeks) and BAPN (150 mg/kg/d, the first 2 weeks). (**G**) Schematic of BAPN/Ang II–induced AAA model. (**H**) Representative morphology of aortae at the endpoint. (**I**) Survival curve. (**J**) Incidence of AAA formation. (**K**) Representative H&E and VVG staining of suprarenal abdominal aortae; red arrows indicate elastin degradation. Scale bars: 200 μm; 20 μm (higher-magnification images). Data are presented as mean ± SEM. *P* values were calculated using Mann-Whitney *U* test (**C** and **F**), Mantel-Cox method (**I**), and χ^2^ test (**J**).

To induce AAA, 12-week-old *Tcf7l2^SMKO^* and *Tcf7l2^fl/fl^* mice, both on an *Apoe^–/–^* background, were infused with angiotensin II (Ang II; 1,000 ng/kg/min) for 28 days (Figure 2A). Compared with controls, *Tcf7l2^SMKO^ Apoe^–/–^* mice showed markedly reduced suprarenal abdominal aortic dilation and decreased AAA incidence (Figure 2, B–D). Histological analyses using hematoxylin and eosin (H&E) and Verhoeff–van Gieson (VVG) staining further revealed attenuated luminal dilation, thrombus formation, and elastin degradation in *Tcf7l2^SMKO^ Apoe^–/–^* mice (Figure 2, E and F). No significant differences in systolic blood pressure, body weight, blood cholesterol, and triglyceride levels were observed between genotypes, ruling out systemic factors as confounders (Supplemental Figure 3, A–C). These findings indicate that VSMC-specific deletion of TCF7L2 protects against Ang II–induced AAA formation.

### Smooth muscle cell–specific TCF7L2 deficiency reduces AAA formation and rupture in the β-aminopropionitrile/Ang II–induced AAA model.

We further validated the role of TCF7L2 in AAA pathology using a more severe murine AAA model combining the lysyl oxidase inhibitor β-aminopropionitrile (BAPN; 150 mg/kg/d for the first 2 weeks, which disrupts collagen cross-linking) with Ang II infusion (1,000 ng/kg/min for 4 weeks) (Figure 2G), known to enhance aneurysm formation and rupture risk in wild-type mice. However, *Tcf7l2^SMKO^* mice were significantly resistant to BAPN/Ang II–induced AAA formation and rupture-related mortality (Figure 2, H–J). Histological assessment using H&E and VVG staining confirmed decreased elastin fragmentation and preserved vascular wall structure in *Tcf7l2^SMKO^* mice (Figure 2K). Consistent with previous results, no significant differences in systolic blood pressure or body weight were observed between the groups (Supplemental Figure 3, D and E). These results further support that smooth muscle cell–specific TCF7L2 promotes AAA progression.

### Smooth muscle–specific TCF7L2 deficiency alleviates elastase-induced AAA formation.

To further validate our findings from the Ang II–driven models, we used an elastase-induced AAA model (Supplemental Figure 4A), which directly triggers localized aneurysm formation by enzymatically degrading the elastin-rich medial layer. Consistent with the Ang II results, *Tcf7l2^SMKO^* mice displayed significantly reduced maximal aortic diameters and decreased elastin degradation compared with *Tcf7l2^fl/fl^* controls (Supplemental Figure 4, B–E). Systolic blood pressure and body weight were unchanged between genotypes (Supplemental Figure 3, F and G), supporting a direct, cell-autonomous role of VSMC-specific TCF7L2 in AAA progression.

Collectively, results from 3 independent AAA models consistently demonstrate that VSMC-specific TCF7L2 is a key driver of aneurysm progression, highlighting its potential as a therapeutic target to attenuate pathological vascular remodeling in AAA.

### TCF7L2 regulates gene networks governing ECM turnover and VSMC-ECM adhesion.

**Figure 3:**
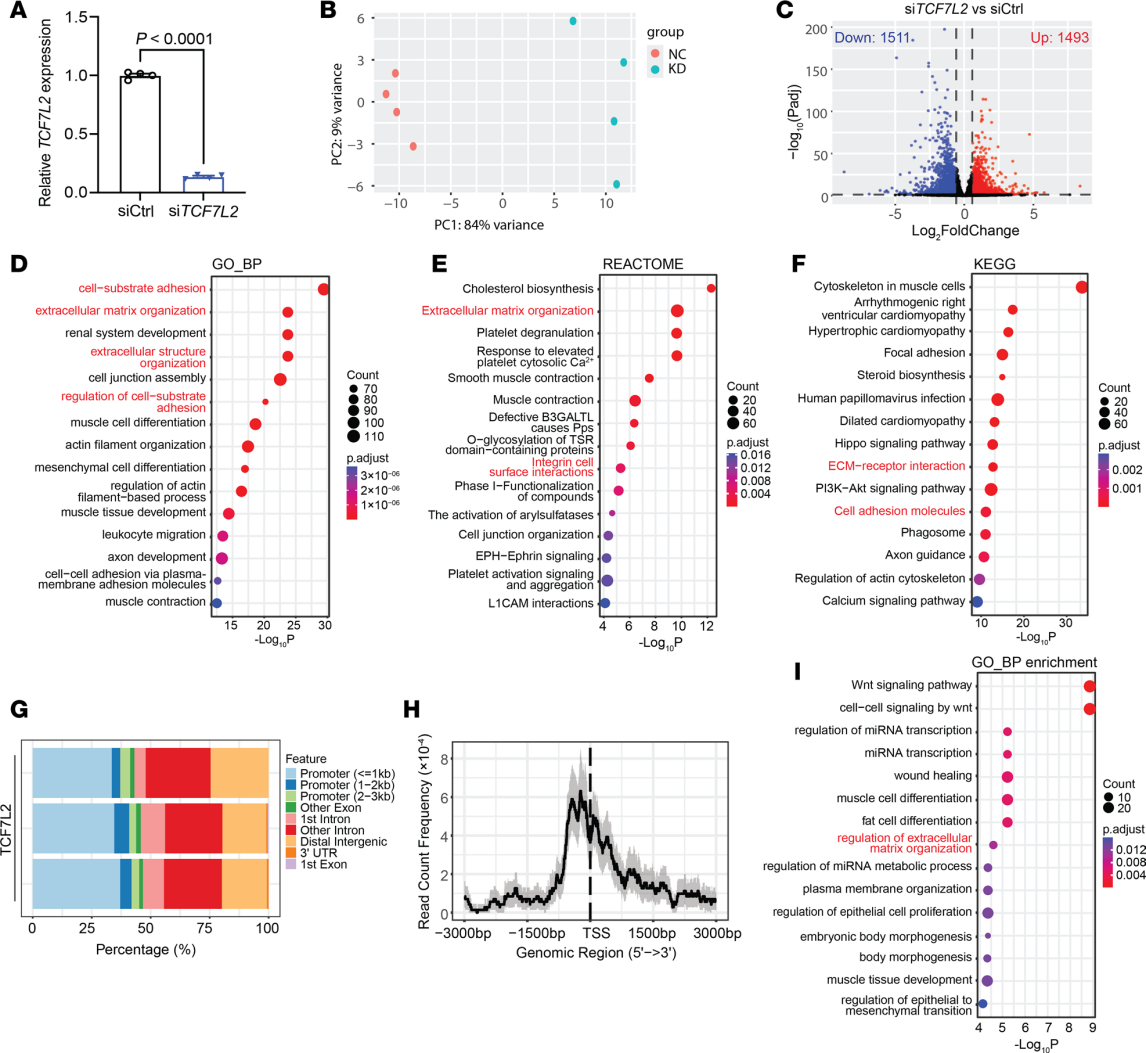
Transcriptomic and epigenetic profiling reveals TCF7L2 as a regulator of ECM turnover and VSMC-ECM adhesion. (**A**–**F**) HASMCs were transfected with 20 nM si*TCF7L2* or siControl for 48 hours, followed by serum starvation in Opti-MEM for 24 hours before RNA isolation for qPCR (**A**) and RNA-seq (**B**–**F**). (**B**) Principal component analysis (PCA). (**C**) Volcano plot showing differentially expressed genes (DEGs); red and blue dots represent significantly upregulated and downregulated genes, respectively. *P*_adj_, adjusted *P* value. (**D**–**F**) Pathway enrichment analysis of DEGs. (**D**) Gene Ontology biological processes (GO_BP). (**E**) Reactome pathways. (**F**) KEGG pathways. (**G**–**I**) For ChIP-seq analysis, HASMCs at about 80% confluence were serum-starved for 24 hours before chromatin isolation. (**G**) Genomic distribution of TCF7L2 ChIP-seq peaks. (**H**) Average TCF7L2 ChIP-seq signal distribution around TSS. (**I**) GO enrichment analysis of TCF7L2-bound genes. Data are presented as mean ± SEM. *P* values were calculated using Student’s *t* test (**A**).

To define the transcriptional programs regulated by TCF7L2 in VSMCs, we performed RNA-seq and chromatin immunoprecipitation sequencing (ChIP-seq) analyses in human aortic smooth muscle cells (HASMCs) following siRNA-mediated TCF7L2 knockdown. qPCR confirmed efficient silencing of *TCF7L2*, with mRNA levels reduced by more than 80% (Figure 3A). Principal component analysis revealed distinct transcriptomic separation between control and knockdown groups, indicating widespread transcriptomic alterations upon TCF7L2 reduction (Figure 3B). Differential gene expression analysis identified 3,004 TCF7L2-responsive genes (|fold change| > 1.5, adjusted *P* value < 0.05), including 1,493 upregulated and 1,511 downregulated genes (Figure 3C).

Functional enrichment analyses was used to interpret the biological implications of these transcriptional changes. Gene Ontology (GO) terms were strongly enriched for ECM organization and cell adhesion (Figure 3D). Reactome (Figure 3E) and KEGG (Figure 3F) pathway analyses further emphasized ECM remodeling, integrin-mediated interactions, and focal adhesion as major pathways regulated by TCF7L2.

To investigate direct transcriptional targets, we performed TCF7L2 ChIP-seq in HASMCs. Genomic distribution analysis revealed that TCF7L2 binding sites were predominantly enriched near transcription start sites (TSSs), consistent with promoter-proximal transcriptional regulation (Figure 3, G and H). GO analysis of genes harboring TCF7L2 binding peaks showed enrichment in Wnt signaling and ECM organization (Figure 3I).

Taken together, these RNA-seq and ChIP-seq data demonstrate that TCF7L2 regulates a coordinated transcriptional network that governs ECM remodeling and VSMC-ECM adhesion, which are crucial processes in pathological vascular remodeling during AAA development.

### TCF7L2 upregulates MMP14 in VSMCs.

To investigate how TCF7L2 contributes to ECM remodeling in AAA, we first examined ECM-related transcriptional changes in the RNA-seq dataset. TCF7L2 knockdown led to marked downregulation of ECM-degrading enzymes, including *MMP14* (also known as MT1-MMP), *ADAMTS2*, and *ADAMTS12*, while genes involved in ECM structural integrity and cellular adhesion, such as *FBN1*, *ITGB1*, and *HAS2*, were upregulated (Supplemental Figure 5A). Because matrix metalloproteinases (MMPs) are key mediators of ECM degradation, we further evaluated expression patterns across the MMP family. Among all MMPs, MMP2 and MMP14 were most abundantly expressed in HASMCs, with MMP14 showing the strongest reduction after TCF7L2 knockdown (Supplemental Figure 5B).

**Figure 4:**
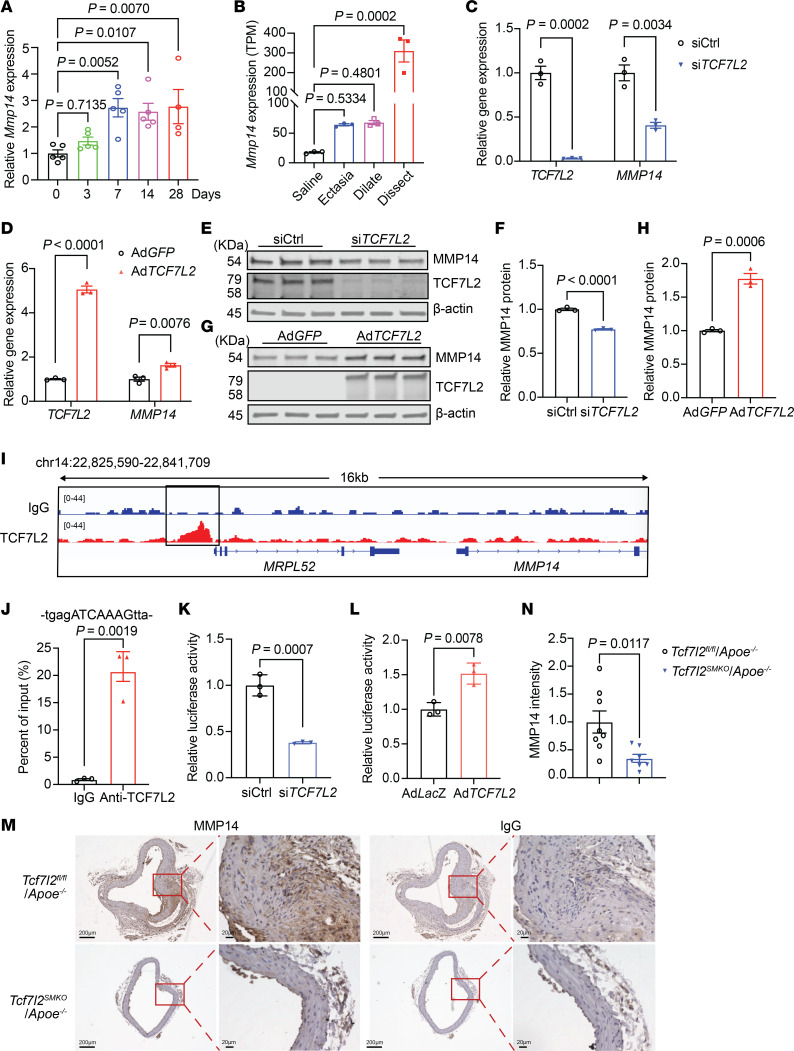
TCF7L2 upregulates MMP14 expression in VSMCs and AAA lesions. (**A**) qPCR analysis of *Mmp14* mRNA levels in abdominal aortae from PCSK9/Ang II–induced AAA mice at days 0, 3, 7, 14, and 28. (**B**) RNA-seq analysis of *Mmp14* expression in abdominal aortae from PCSK9/Ang II–induced AAA mice at day 14, stratified into saline control, ectasia (maximal diameter < 1.2 mm), dilation (≥1.2 mm without dissection), and dissection (presence of intramural hemorrhage). (**C**–**H**) HASMCs were transfected with 20 nM si*TCF7L2* and siControl (**C**, **E**, and **F**) or 20 MOI Ad*TCF7L2* and Ad*GFP* (**D**, **G**, and **H**) for 48 hours, followed by serum starvation in Opti-MEM for 24 hours. mRNA levels of *TCF7L2* and *MMP14* (**C** and **D**) and protein abundance of TCF7L2 and MMP14 (**E**–**H**) were determined from 3 independent experiments. (**I**) Normalized ChIP-seq reads of TCF7L2 in the genomic region upstream of the *MMP14* gene are shown in the IGV image. (**J**) ChIP-qPCR quantification of TCF7L2 binding at the predicted motif upstream of *MMP14*. (**K** and **L**) Dual-luciferase reporter assays in HASMCs pretreated with 20 nM si*TCF7L2* and siControl (**K**) or 20 MOI Ad*TCF7L2* and Ad*GFP* (**L**) for 24 hours, followed by transfection of a minimal CMV luciferase reporter containing the TCF7L2 ChIP-seq–identified upstream regulatory fragment of MMP14. Luciferase activity was measured 24 hours after reporter transfection. (**M**) Representative images of immunohistochemical staining of MMP14 of suprarenal abdominal aortae in the Ang II–induced AAA model. Scale bars: 200 μm; 20 μm (higher-magnification images). (**N**) Quantification of MMP14 staining intensity. *P* values were calculated using 1-way ANOVA followed by Tukey’s post hoc analysis for **A** and **B** or Student’s *t* test for **C**–**N**.

Given the established role of MMP14 in ECM degradation and its critical function in activating MMP2 (17), we next assessed whether its expression changes during AAA progression. In the PCSK9/Ang II mouse model, abdominal aortic *Mmp14* expression increased significantly by day 7 and remained elevated through day 28 (Figure 4A). Analysis of an independent published RNA-seq dataset (18) revealed that *Mmp14* expression correlated with aneurysm severity, increasing stepwise from ectasia (maximal diameter < 1.2 mm) to dilation (≥1.2 mm without dissection) to dissection (intramural hemorrhage) stages at day 14 after Ang II infusion (Figure 4B). These findings suggest that MMP14 is progressively induced during AAA progression.

In vitro validation in HASMCs demonstrated that *TCF7L2* knockdown significantly reduced both MMP14 mRNA and protein levels, whereas *TCF7L2* overexpression led to elevated MMP14 expression (Figure 4, C–H). To elucidate whether TCF7L2 directly regulates *MMP14* transcription, we analyzed our TCF7L2 ChIP-seq dataset generated from HASMCs, identifying a prominent TCF7L2 binding peak located approximately 7 kb upstream of the *MMP14* TSS, which we validated by ChIP-qPCR (Figure 4, I and J). To confirm the functional activity of this regulatory element, we cloned the binding peak region into a luciferase reporter (Supplemental Figure 9) and measured reporter activity after modulating TCF7L2. TCF7L2 knockdown reduced, whereas TCF7L2 overexpression increased, enhancer-driven luciferase activity (Figure 4, K and L), supporting a direct activating role of TCF7L2 at this *MMP14* regulatory element. Although this peak overlaps the promoter region of the neighboring gene *MRPL52*, modulation of TCF7L2 did not alter *MRPL52* expression (Supplemental Figure 8, A and B), indicating that TCF7L2 binding at this site selectively regulates *MMP14*.

Finally, immunohistochemistry of aortic tissues from Ang II–induced AAA mice showed that MMP14 protein levels were substantially reduced in *Tcf7l2^SMKO^ Apoe^–/–^* mice compared with controls (Figure 4, M and N), confirming TCF7L2-dependent regulation of MMP14 in vivo. Together, these results identify MMP14 as a direct transcriptional target of TCF7L2 and support a model in which TCF7L2 promotes ECM degradation in AAA at least in part by upregulating MMP14 expression in VSMCs.

### TCF7L2 represses TIMP3 and promotes proteolytic activity.

**Figure 5:**
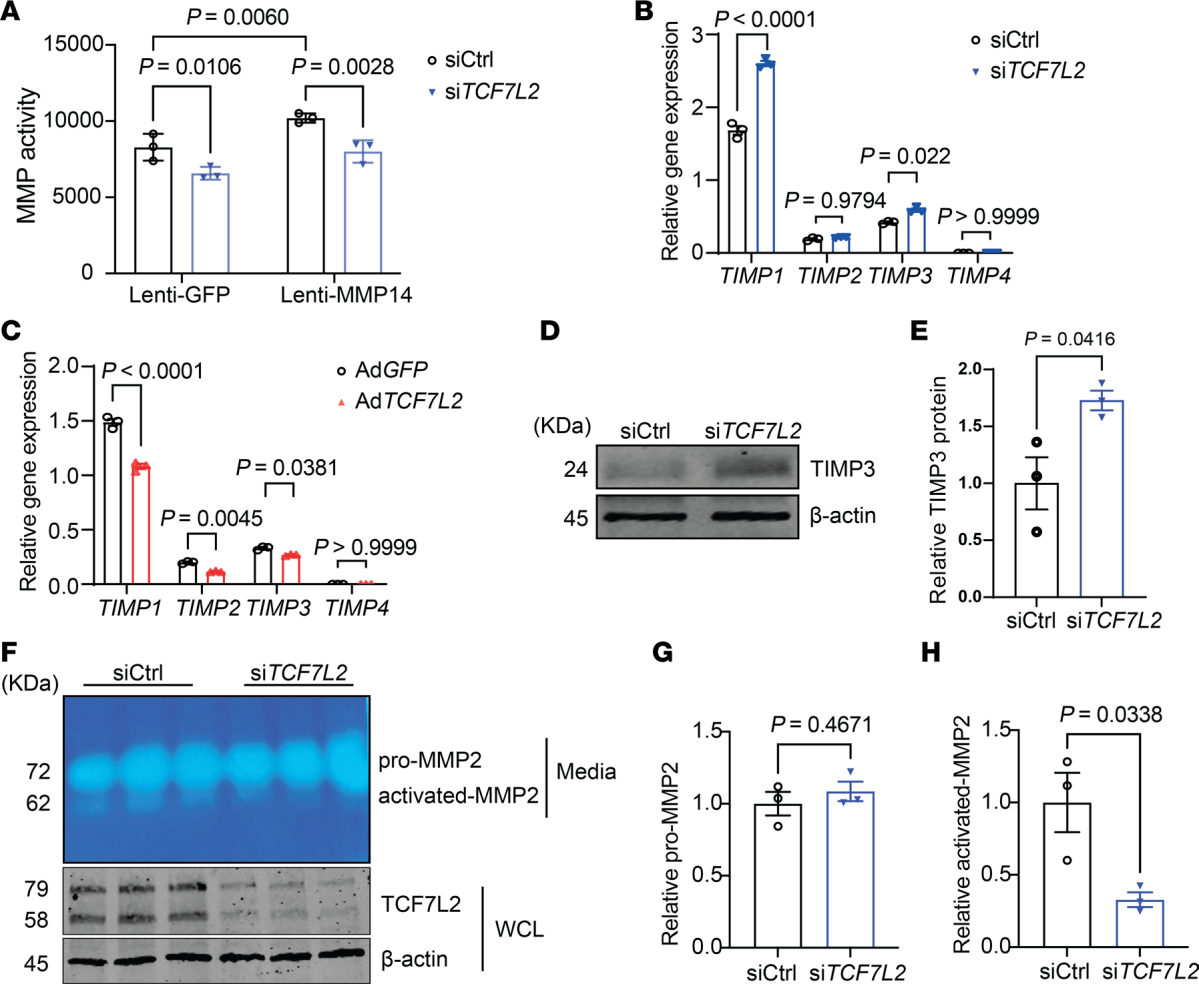
TCF7L2 promotes MMP activity by upregulating MMP14 and repressing TIMP3. (**A**) For MMP activity assays, HASMCs were first transfected with 20 nM si*TCF7L2* or siControl for 48 hours, then infected with either Lenti-GFP or Lenti-MMP14 for 24 hours, and subsequently serum-starved in Opti-MEM for 48 hours. Conditioned media were collected, and total MMP activity was assessed using a fluorometric assay. (**B**–**E**) HASMCs were transfected with 20 nM si*TCF7L2* and siControl or 20 MOI Ad*TCF7L2* and Ad*GFP* for 48 hours, followed by serum starvation in Opti-MEM for 24 hours, and mRNA levels of *TCF7L2* and *TIMPs* (**B** and **C**) and protein abundance of TIMP3 (**D** and **E**) were determined from 3 independent experiments. (**F**–**H**) HASMCs were transfected with 20 nM si*TCF7L2* and siControl, followed by serum starvation in Opti-MEM for 24 hours; gelatin zymography was performed to assess the levels of pro-MMP2 and activated MMP2 in the conditioned media; and Western blot was used to evaluate TCF7L2 knockdown efficiency in whole-cell lysates. Data are presented as mean ± SEM. *P* values were calculated using 2-way ANOVA followed by Holm-Šidák post hoc analysis for **A**–**C** or Student’s *t* test for **E**, **G**, and **H**.

To assess how TCF7L2 influences pericellular proteolysis, we first measured total MMP activity in conditioned media. Overexpression of MMP14 increased MMP activity, whereas *TCF7L2* knockdown markedly reduced it. Notably, reduced MMP activity persisted even when MMP14 was overexpressed (Figure 5A), indicating that TCF7L2 acts through additional downstream targets beyond MMP14 alone.

Given that pericellular MMP activity is also governed by endogenous inhibitors, we next examined the expression of tissue inhibitors of metalloproteinases (TIMPs). Among the 4 TIMP family members, TIMP1, TIMP2, and TIMP3 were predominantly expressed in HASMCs. TCF7L2 knockdown markedly increased *TIMP1* and *TIMP3* mRNA levels, whereas TCF7L2 overexpression suppressed *TIMP1*, *TIMP2*, and *TIMP3* (Figure 5, B and C). TIMP1 is primarily soluble, whereas TIMP3 is ECM bound and inhibits pericellular MMPs, particularly MMP2 and MMP9 (19). Consistent with its transcriptional upregulation, TIMP3 protein abundance was also significantly increased following TCF7L2 knockdown (Figure 5, D and E).

To validate this finding in vivo, we analyzed aortic tissues from *Tcf7l2^SMKO^* and floxed control mice subjected to Ang II–induced AAA. Consistent with our in vitro data, *Timp3* mRNA expression was significantly increased, whereas *Mmp9* mRNA expression was decreased (Supplemental Figure 6, A–F). Immunohistochemical staining further confirmed elevated TIMP3 protein abundance in the vascular wall of *Tcf7l2^SMKO^* mice (Supplemental Figure 6G), supporting the in vivo relevance of TCF7L2-mediated regulation of proteolytic activity and ECM remodeling.

Because MMP14 activates pro-MMP2 and TIMP3 inhibits MMP2 activity, we next evaluated MMP2 activation. Gelatin zymography revealed that TCF7L2 knockdown markedly decreased active MMP2 levels in HASMC culture medium (Figure 5, F–H), which was further supported by reduced MMP2 and MMP9 activities in abdominal aorta aneurysmal tissue extracts from *Tcf7l2^SMKO^* mice compared with the control group (Supplemental Figure 7).

In summary, these findings indicate that TCF7L2 promotes ECM degradation by enhancing MMP activity through coordinated upregulation of MMP14 and repression of TIMP3, thereby sustaining proteolytic remodeling in AAA.

### TCF7L2 downregulates ITGB1 to weaken VSMC-ECM adhesion.

**Figure 6:**
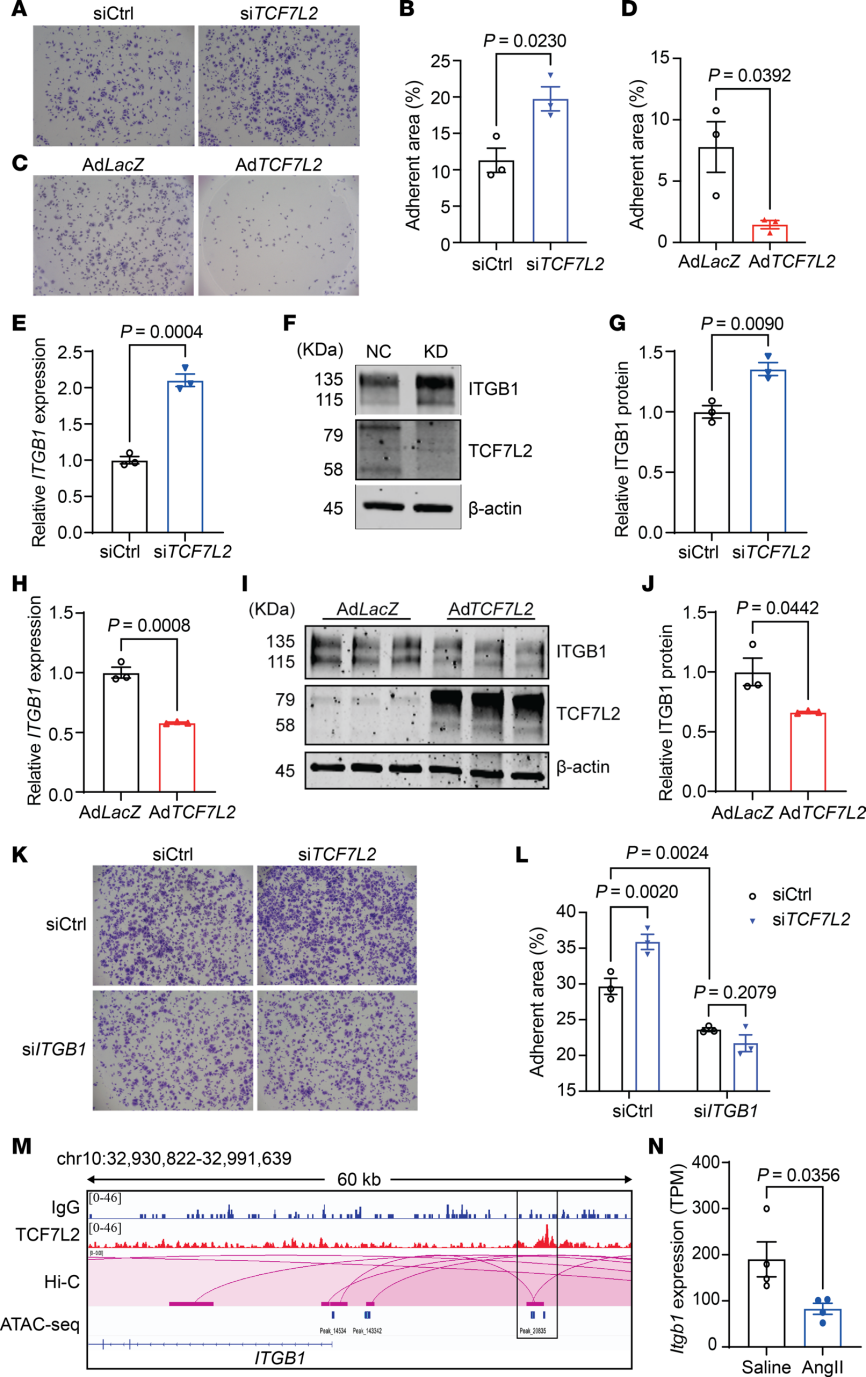
TCF7L2 represses ITGB1 to disrupt VSMC-ECM adhesion. (**A**–**D**) HASMCs were transfected with 20 nM si*TCF7L2* or siControl (**A** and **B**) or infected with 20 MOI Ad*TCF7L2* or AdGFP (**C** and **D**) for 48 hours, followed by serum starvation in Opti-MEM for 24 hours. Cells were then plated onto collagen I–coated wells and allowed to adhere at 37°C for 90 minutes. Adherent cells were visualized by crystal violet staining (**A** and **C**) and quantified (**B** and **D**). The original magnification of the images was 4×. (**E**–**J**) Under the same transfection or infection conditions as above, mRNA levels (**E** and **H**) and protein abundance (**F**, **G**, **I**, and **J**) of ITGB1 were determined from 3 independent experiments. (**K** and **L**) HASMCs were cotransfected with 20 nM si*TCF7L2* or siControl and either 20 nM si*ITGB1* or control siRNA for 48 hours, followed by adhesion assay as in **A**–**D**. The original magnification of the images was 4×. (**M**) IGV browser view of ChIP-seq signal tracks showing TCF7L2 and IgG binding at the human *ITGB1* locus, with aligned Hi-C (ENCSR797MWY) and ATAC-seq (ENCFF719YRB) profiles. (**N**) Bulk RNA-seq analysis of *Itgb1* expression in abdominal aortae from PCSK9/Ang II–induced AAA mice versus saline controls at day 14. Data are presented as mean ± SEM. *P* values were calculated using Student’s *t* test for **B**, **D**, **E**, **G**, **H**, **J** and **N**, and 2-way ANOVA followed by Holm-Šidák post hoc analysis for **L**.

Pathway enrichment analysis of our RNA-seq data identified cell adhesion as another major biological process regulated by TCF7L2 in HASMCs. Given this, we first evaluated VSMC adhesion to collagen I–coated substrates. TCF7L2 knockdown significantly increased VSMC adhesion (Figure 6, A and B), whereas adenoviral *TCF7L2* overexpression markedly reduced adhesion (Figure 6, C and D), indicating that TCF7L2 negatively regulates VSMC-ECM adhesion.

Among adhesion-related genes, integrin β_1_ (*ITGB1*), a key mediator of VSMC-ECM interactions, was strongly upregulated upon TCF7L2 knockdown. This increase was confirmed at both mRNA and protein levels (Figure 6, E–G). Conversely, adenovirus-mediated overexpression of *TCF7L2* significantly suppressed *ITGB1* expression (Figure 6, H–J).

To determine whether ITGB1 mediates TCF7L2-dependent regulation of VSMC-ECM adhesion, we next performed ITGB1 knockdown. Silencing ITGB1 reduced VSMC adhesion and abrogated the adhesion increase caused by TCF7L2 knockdown (Figure 6, K and L), indicating that ITGB1 is required for TCF7L2-dependent regulation of adhesion.

To investigate the regulatory mechanism, ChIP-seq analysis revealed a prominent TCF7L2-binding peak upstream of the *ITGB1* locus that overlapped with regions of accessible chromatin detected by ATAC-seq. Hi-C interaction maps further suggested looping between this distal regulatory element and the *ITGB1* promoter (Figure 6M), supporting direct transcriptional repression of *ITGB1* by TCF7L2.

Finally, we examined *Itgb1* expression in abdominal aortae from the PCSK9/Ang II–induced AAA mouse model. Compared with saline-treated controls, abdominal aortae from Ang II–treated mice exhibited significantly reduced *Itgb1* expression (Figure 6N), consistent with the repressive effect of TCF7L2 observed in vitro and indicative of impaired VSMC-ECM adhesion during AAA development.

Taken together, these data establish ITGB1 as a direct downstream effector of TCF7L2 and demonstrate that TCF7L2-dependent repression of ITGB1 compromises VSMC-ECM adhesion, thereby promoting AAA progression.

## Discussion

In this study, we integrate human genetic evidence with in vivo and in vitro functional analyses to establish TCF7L2 as a critical regulator of AAA pathogenesis. SMR analysis demonstrated that elevated aortic TCF7L2 expression is causally associated with increased AAA risk, and smooth muscle–specific deletion of TCF7L2 consistently protected against AAA formation across 3 independent mouse models. Mechanistically, TCF7L2 drives pathological ECM remodeling by enhancing MMP activity through the induction of MMP14 and suppression of TIMP3, while simultaneously impairing VSMC-ECM adhesion by repressing ITGB1. Together, these findings identify TCF7L2 as a key transcriptional regulator linking genetic susceptibility to pathological ECM remodeling in AAA.

A strong genetic component to AAA has been supported by family aggregation and twin studies (20, 21), and GWAS have identified numerous non-coding susceptibility loci for vascular diseases (10, 22–25). Among these, TCF7L2 stands out as a shared risk locus for both AAA and thoracic aortic aneurysm (11, 12), suggesting a broader role in vascular homeostasis and aneurysm susceptibility. Because most disease-associated variants reside in non-coding regions, integrative approaches are required to infer functional relevance (26). By combining GWAS and aortic eQTL data using SMR, a method that assesses causal relationships between gene expression and disease risk while limiting confounding from linkage disequilibrium and pleiotropy (27), our study provides evidence that increased aortic TCF7L2 expression likely contributes causally to AAA risk. The protective effect observed in 3 distinct AAA models with VSMC-specific TCF7L2 depletion further supports a direct role of TCF7L2 in aneurysm development, independent of systemic parameters.

TCF7L2 is a major transcriptional effector of canonical Wnt/β-catenin signaling, which is extensively studied in development and cancer (28). Increasing evidence implicates aberrant Wnt activation in aortic aneurysm pathology. Human AAA tissues display upregulation of WNT ligands such as WNT2, WNT5A, and WNT5B (29), together with downregulation of the endogenous inhibitor SOST (30). Restoring SOST expression attenuates experimental AAA by preserving ECM integrity, reducing elastin degradation, and limiting inflammation (30). Similar Wnt/β-catenin activation has been reported in thoracic aortic aneurysm and aortic dissection (31, 32), pointing to a broader involvement of Wnt/β-catenin signaling in aortic wall degeneration.

Within the TCF/LEF family, TCF7L2 and TCF7L1 are the predominant isoforms expressed in human abdominal aorta, whereas TCF7 and LEF1 are minimally detected. Structural homology among TCF/LEF family members suggests potential functional redundancy, consistent with recent work showing that TCF7L1 promotes AAA through regulating VSMC phenotypic switching (34). However, the vascular functions of TCF7L2 remain incompletely defined and sometimes contradictory. Studies have reported that TCF7L2 inhibits VSMC apoptosis and promotes proliferation (35), whereas others indicate that it suppresses proliferation and enhances differentiation (36, 37). Additional evidence suggests that TCF7L2 may promote VSMC apoptosis by repressing anti-apoptotic BCL2 (12). These discrepancies highlight the complex, context-dependent role of TCF7L2 in vascular remodeling and underscore the need for mechanistic dissection in disease-relevant settings such as AAA.

Our integrated RNA-seq and ChIP-seq analyses provide insight into how TCF7L2 influences VSMC behavior in AAA. Rather than acting through a single effector, TCF7L2 regulates a coordinated set of gene programs involved in ECM turnover, cell-matrix adhesion, and cytoskeletal organization. These interconnected processes are essential for preserving the structural integrity of the aortic media.

Excessive ECM degradation is a hallmark of AAA and contributes directly to medial weakening and rupture (38, 39). Among ECM degradation enzymes, MMP14 is a critical initiator of pericellular matrix degradation and a well-established activator of pro-MMP2 (40). Our findings identify TCF7L2 as an upstream regulator of this axis. TCF7L2 directly induces MMP14 transcription and simultaneously represses TIMP3. This coordinated regulation shifts the protease-inhibitor balance toward enhanced matrix proteolysis, consistent with the marked reduction in active MMP2 and total MMP activity observed following TCF7L2 knockdown. Importantly, these biochemical changes correspond to our in vivo findings, in which VSMC-specific *Tcf7l2* deletion attenuated elastin degradation in the aortic media, as demonstrated by VVG staining. Together, these results suggest that TCF7L2 enhances the proteolytic milieu of the aortic wall, thereby facilitating ECM breakdown during AAA progression.

Stable interactions between VSMCs and the ECM are essential for maintaining medial structure and distributing mechanical stress across the aortic wall (41). ITGB1 is a principal mediator of VSMC-ECM adhesion and plays an important role in maintaining vessel wall resilience (42). Studies show that ITGB1 upregulation preserves aortic wall integrity and reduces susceptibility to aneurysmal dilation (43), whereas ITGB1 deficiency impairs maintenance of vessel structure (44). Consistent with these observations, our data demonstrate that TCF7L2 suppresses VSMC adhesion to collagen I and concurrently downregulates ITGB1 expression. Silencing ITGB1 abrogated the adhesion-promoting effect observed in TCF7L2-deficient cells, indicating that ITGB1 is a key downstream effector mediating this phenotype. Thus, TCF7L2 simultaneously enhances ECM proteolysis and weakens VSMC-ECM adhesion, two synergistic pathological processes that compromise medial integrity and heighten susceptibility to aneurysm formation.

Several limitations should be acknowledged. First, although our results support a TCF7L2–MMP14/TIMP3–ECM degradation axis and ITGB1-VSMC adhesion axis, direct in vivo rescue experiments will be required to conclusively demonstrate that TCF7L2 transcriptional control drives ECM remodeling in the aortic wall. Second, the specific risk allele at the *TCF7L2* locus identified in our genetic analyses has not been functionally characterized. Determining whether this variant directly alters enhancer activity, chromatin accessibility, or transcription factor binding will require future allele-specific perturbation approaches such as CRISPR editing and reporter assays. Third, therapeutic translation remains a key challenge. Although our data suggest TCF7L2 as a potential therapeutic target, strategies that allow selective and safe modulation of TCF7L2 activity in vascular tissues remain to be developed. The development of small-molecule inhibitors or targeted gene-modulating tools will be essential for evaluating the translational feasibility of TCF7L2 inhibition in AAA.

In summary, this study integrates human genetic evidence, vascular smooth muscle–specific gene deletion, and mechanistic validation to identify TCF7L2 as a key regulator of AAA pathogenesis. Smooth muscle cell–specific TCF7L2 deficiency consistently protected against aneurysm formation across 3 independent mouse models, establishing its pathogenic role in vivo. Mechanistically, TCF7L2 drives maladaptive ECM remodeling by enhancing the MMP14/TIMP3–MMP2 degradative axis and repressing ITGB1, thereby weakening VSMC-ECM adhesion. Together, these findings identify TCF7L2 as a molecular link between genetic susceptibility and structural degeneration of the aortic wall and highlight it as a promising therapeutic target to limit AAA progression.

## Methods

Additional details may be found in supplemental materials.

### Sex as a biological variable

This study used human aortic smooth muscle cells derived from both male and female donors. In mouse experiments, only male mice were used, because the Cre transgene in *Myh11*-CreER^T2^ mice is Y chromosome–linked, precluding its use in females.

### Materials and reagents

Antibodies against TCF7L2 (catalog 2569), integrin β_1_ (catalog 4706), and β-actin (catalog 3700) were purchased from Cell Signaling Technology. The MMP14 antibody (ab51074) was obtained from Abcam, and the TIMP3 antibody (10858-1-AP) was purchased from Proteintech. BAPN (A3134) and elastase (E1250) were obtained from Sigma-Aldrich. Ang II (catalog 4006473) was obtained from Bachem. Collagen I (A1064401) was purchased from Thermo Fisher Scientific.

### Summary data–based Mendelian randomization

Summary data–based Mendelian randomization (SMR) analysis was performed using the SMR software package (v1.3.1) as previously described (27). GWAS summary statistics of AAA were obtained from the AAAgen Consortium meta-analysis, including 39,221 cases and 1,086,107 controls (11), and *cis*-eQTL data for aortic tissue were retrieved from the GTEx v8 database. Individual-level genotype data were retrieved from the European reference panels from the 1000 Genomes Project for linkage disequilibrium estimation. We adopted the following settings in SMR: the window centered around the probe to select *cis*-eQTL/methylation quantitative trait loci (mQTL) was set as 500 kbp; the *P* value threshold to select the top associated eQTL/mQTL for the SMR test was set as 5.0 × 10^–6^; and the threshold of QTL *P* value to select QTLs for the HEIDI test was set as 1.57 × 10^–3^, equivalent to a χ^2^ value of 10. Multiple-testing correction was performed using the Bonferroni method, with a significance threshold of FDR < 0.05. The statistical analyses and visualizations were conducted using SMR (v1.3.1) and R (ggplot2 package) (27).

### Animals and murine AAA models

*Tcf7l2^fl/fl^* mice were generated by the University of Michigan Transgenic Core using JM8.F6 embryonic stem (ES) cells. These ES cells carried the *Tcf7l2^tm1a(EUCOMM)Wtsi^* allele, in which *loxP* sites flank exon 5 of the *Tcf7l2* gene, and were obtained from the International Mouse Phenotyping Consortium. To achieve SMC-specific *Tcf7l2* deletion, *Tcf7l2^fl/fl^* mice were crossed with *Myh11*-CreER^T2^ transgenic mice (The Jackson Laboratory, strain 019079). These mice were further crossed with *Apoe^–/–^* mice (The Jackson Laboratory, strain 002052) to generate *Myh11*-CreER^T2^ *Tcf7l2^fl/fl^* *Apoe^–/–^* mice. As *Myh11*-CreER^T2^ is Y chromosome–linked (45), only male mice were used in this study. Tamoxifen (Sigma-Aldrich, T5648; 75 mg/kg/d) was administered by oral gavage for 5 consecutive days to induce *Tcf7l2* deletion in SMCs (46). After a 9-day washout period, AAA was induced using established protocols (16).

#### Ang II–induced AAA model.

The Ang II–induced AAA model was established as previously described (47). Briefly, 12-week-old male *Tcf7l2^SMKO^* *Apoe^–/–^* and *Tcf7l2^fl/fl^* *Apoe^–/–^* mice were used. Baseline body weight and blood pressure were measured before treatment, with blood pressure assessed using a tail-cuff system (Visitech Systems, BP-2000 Series II). Mice were subcutaneously implanted with osmotic minipumps (Alzet, model 2004) to continuously infuse Ang II (1,000 ng/kg/min) for 4 weeks. At the study endpoint, final body weight and blood pressure were measured before euthanasia.

#### BAPN/Ang II–induced AAA model.

The BAPN/Ang II–induced AAA model was established as previously described (48). Briefly, 16-week-old male *Tcf7l2^SMKO^* and *Tcf7l2^fl/fl^* mice were used. The experimental procedure followed the Ang II–induced AAA model, with the addition of BAPN administration. BAPN was prepared in sterile saline at a concentration of 150 mg/kg/d and delivered for 14 days via a separate osmotic minipump (Alzet, model 2002). During the implantation procedure, both the BAPN- and Ang II–infusing pumps were placed subcutaneously.

#### Elastase-induced AAA model.

The elastase-induced AAA model was established as previously described (49). Twelve- to fourteen-week-old male *Tcf7l2^SMKO^* and *Tcf7l2^fl/fl^* mice were used. Baseline body weight and blood pressure were measured before surgery. Mice were anesthetized via intraperitoneal injection of ketamine (100 mg/kg) and xylazine (10 mg/kg). The infrarenal abdominal aorta was carefully isolated, and a sterile strip was placed underneath for support. A 3 mm × 1.5 cm sterile cotton strip pre-soaked with about 30 μL elastase (44 U/mL; Sigma-Aldrich, E1250) was wrapped around the aorta and incubated for 30 minutes. After incubation, the aorta was rinsed thoroughly with sterile saline before suturing. Mice were monitored postoperatively, and after 14 days, final body weight and blood pressure were measured before euthanasia, AAA characterization, and tissue collection.

For the *Pcsk9*/Ang II–induced AAA model in C57BL/6J mice, 8-week-old male mice were intravenously injected with adeno-associated virus (serotype 8) containing murine *Pcsk9* D377Y (Penn Vector Core; a gain-of-function mutation) at a dose of 2 × 10^11^ genome copies per mouse while simultaneously being fed a Western diet (50). After 2 weeks, Ang II (1,000 ng/kg/min) was subcutaneously infused via a minipump (Alzet, model 2004). Aortic tissues were harvested at defined time points (days 0–28) for gene expression analysis.

Mice were euthanized 2 weeks after elastase perfusion or 4 weeks after Ang II infusion. Blood was collected via ventricular puncture, followed by systemic perfusion through the left ventricle using saline to flush out remaining blood and then 10% neutral-buffered formalin in PBS to preserve the tissue samples. The abdominal aortae were carefully dissected for ex vivo morphological assessment. For AAA evaluation, the maximum external diameter of the infrarenal aorta (in the elastase model) or suprarenal aorta (in the Ang II, BAPN/Ang II, and *Pcsk9*/Ang II models) was measured. AAA was defined as a maximum diameter at least 50% greater than that of adjacent non-aneurysmal segments (16).

#### Plasma total cholesterol and triglyceride measurement.

Blood samples were collected from mice and centrifuged at 4°C, approximately 1,500*g*, for 20 minutes, to obtain plasma. Plasma total cholesterol (TC) and triglyceride (TG) levels were measured using commercial assay kits (Fujifilm Wako, 999-02601 for TC and 632-50991 for TG) according to the manufacturer’s instructions.

### Histology

Murine aortic samples were fixed in 10% neutral-buffered formalin for at least 24 hours. Paraffin embedding and staining were performed by the Unit for Laboratory Animal Medicine (ULAM) Pathology Core at the University of Michigan. Serial 5 μm sections were prepared for H&E staining, VVG/Masson’s trichrome–Verhoeff (MTC-V) staining (elastin assessment), and immunohistochemistry.

Immunohistochemistry was performed using the Rabbit Specific HRP/DAB Detection IHC Kit (Abcam, ab64261) according to the manufacturer’s instructions. Briefly, paraffin-embedded tissue sections were deparaffinized, rehydrated, and treated with hydrogen peroxide block for 10 minutes to quench endogenous peroxidase activity. After antigen retrieval in citrate buffer at 92°C for 20 minutes, sections were washed and incubated with 5% goat serum for 1 hour to block nonspecific binding. Sections were then incubated with MMP14 primary antibody (1:100) or matched-concentration IgG control at 37°C for 1 hour, followed by overnight incubation at 4°C. The next day, sections were incubated with biotinylated goat anti-rabbit secondary antibody and streptavidin-peroxidase, followed by DAB chromogen development. Slides were counterstained with hematoxylin, dehydrated through 70%–100% graded ethanol, cleared, and mounted with a resin-based medium.

### Cell culture

Human aortic smooth muscle cells (HASMCs; CC-2571) were obtained from Lonza and cultured in SMC growth medium 2 (Lonza, CC-3182) supplemented with 5% fetal bovine serum (Lonza) and 1% penicillin/streptomycin solution (Gibco, 5140122) at 37°C, 5% CO_2_, in a humidified cell culture incubator. HASMCs from passages 4 to 8 were used for experiments. Before experiments, HASMCs were serum-starved for 24 hours in Opti-MEM I (Gibco, 31985070).

#### siRNA transfection.

HASMCs were transfected with 20 nM si*TCF7L2* (D-003816-01, GAUGGAAGCUUACUAGAUU) and 20 nM si*ITGB1* (L-004506-00-0005) or 20 nM Silencer Select Negative Control siRNA (siControl; D-001206-14, UAAGGCUAUGAAGAGAUAC) from Horizon Discovery for 48 hours, using jetPRIME transfection reagent (Polyplus, 55-133) according to the manufacturer’s protocol.

#### Adenovirus infection.

HASMCs were infected with adenovirus expressing human TCF7L2 (Ad*TCF7L2*) or control adenovirus expressing GFP or LacZ (Ad*GFP*/Ad*LacZ*). These adenoviral vectors were generated and validated as previously described (12). Cells were infected at the indicated MOI in complete growth medium for 24 hours, after which the medium was replaced with Opti-MEM. Experiments were performed 48 hours after infection. Overexpression efficiency was confirmed by qPCR and Western blot.

#### Lentivirus infection.

HASMCs were infected with a lentivirus expressing human MMP14 (Lenti-MMP14) or a control lentivirus expressing GFP (Lenti-GFP), provided by the laboratory of Stephen Weiss at the University of Michigan and previously described (51). Lentiviral infection was performed using the same procedure as described above for adenovirus infection.

### Total RNA isolation, reverse transcription, and qPCR

Total RNA was isolated from HASMCs using the RNeasy Mini Kit (QIAGEN, 74106) according to the manufacturer’s protocol. RNA concentration and purity were evaluated by a NanoDrop Spectrophotometer (Thermo Fisher Scientific, ND-ONE-W). cDNA synthesis was performed with SuperScript III Reverse Transcriptase (Invitrogen, 18080051). Quantitative real-time PCR (qPCR) was carried out using 2× SYBR Green Fast qPCR Mix (ABclonal, RM21203) on a CFX Connect real-time PCR system (Bio-Rad Laboratories Inc.). Gene expression data were normalized to the housekeeping gene *PPIA*, and relative gene expression levels were calculated using the 2^−ΔΔCt^ method (52). The PCR primers used in this study are listed in Supplemental Table 1.

### RNA-seq

RNA library preparation and sequencing were performed by the Advanced Genomics Core at the University of Michigan. Briefly, RNA quality was assessed using a Bioanalyzer (Agilent), and libraries were prepared with the NEBNext Ultra RNA Library Prep Kit (New England Biolabs, E7770). Sequencing was conducted on an Illumina NovaSeq 6000, generating 101 bp paired-end reads. Raw reads were trimmed with Cutadapt (https://cutadapt.readthedocs.io/), assessed for quality using FastQC (https://www.bioinformatics.babraham.ac.uk/projects/fastqc/) and Fastq Screen (https://www.bioinformatics.babraham.ac.uk/projects/fastq_screen/), and aligned to the human reference genome (GRCh38) with STAR (https://github.com/alexdobin/STAR; commit ID b1edc12), following ENCODE guidelines. Gene expression was quantified using RSEM (https://deweylab.github.io/RSEM/), producing raw counts and TPM values. Differential expression analysis was performed using DESeq2, with additional filtering in limma (|fold change| > 1.5, adjusted *P* value < 0.05). Functional enrichment analysis of differentially expressed genes was conducted using clusterProfiler (GO, KEGG) and ReactomePA (Reactome pathways). Redundant GO terms were consolidated (similarity cutoff = 0.7). Gene set enrichment analysis (GSEA) was performed using GSEA software.

#### RNA-seq data sources for Mmp14 expression.

Bulk RNA-seq data for Figure 4B were obtained from previously published studies performed by members of our laboratory (18). RNA-seq profiles were derived from suprarenal abdominal aorta collected at day 14 and stratified by AAA severity (saline, ectasia, dilation, dissection) (GSE285959) (18).

Bulk RNA-seq data of human abdominal aortae were obtained from a dataset previously published by members of our laboratory (16) and used to assess TCF/LEF family gene expression.

### ChIP assay and ChIP-seq

ChIP assay was performed using the SimpleChIP Enzymatic Chromatin IP Kit (Cell Signaling Technology, 9003) following the manufacturer’s protocol. Briefly, HASMCs were cross-linked with 1% final concentration of formaldehyde, quenched with glycine, and lysed. Chromatin was fragmented using micrococcal nuclease digestion and sonication. Sheared chromatin was immunoprecipitated with an anti-TCF7L2 antibody (1:50) or matched-concentration IgG control, followed by incubation with protein G magnetic beads. After sequential washes, chromatin was eluted, cross-links were reversed at 65°C, and DNA was purified for qPCR analysis. The primers used in this assay are listed in Supplemental Table 1.

For ChIP-seq, library preparation and sequencing were performed by the Advanced Genomics Core at the University of Michigan. Approximately 2 ng of immunoprecipitated chromatin per sample was used for library preparation with the Illumina NEBNext Ultra II DNA Library Prep Kit (New England Biolabs, E7645). Sequencing was conducted on a NovaSeq X platform (151 bp paired-end reads, ~50 million reads per sample). Reads were processed using FastQC and aligned to the GRCh38 genome with Bowtie 2 (http://bowtie-bio.sourceforge.net/bowtie2/). Peak calling was performed with MACS2 (https://github.com/macs3-project/MACS; commit ID d44cf4e). with the following parameters: -f BAMPE -g hs -B -q 0.01; and reproducibility was assessed via irreproducible discovery rate analysis (threshold = 0.05). Peak visualization and annotation, including TSS distance analysis, were conducted using deepTools (53) and ChIPseeker (54).

### Public chromatin profiling data

Publicly available human aortic Hi-C and assay for transposase-accessible chromatin using sequencing (ATAC-seq) datasets were obtained from the ENCODE database (55, 56). The Hi-C dataset (ENCSR797MWY) was generated from abdominal aortic tissue of a 41-year-old female donor using DNase digestion and was used to assess chromatin interactions around the *ITGB1* locus. The ATAC-seq dataset (ENCFF719YRB) was derived from the thoracic aortic tissue of a 54-year-old male donor and provided chromatin accessibility information in the same genomic region. Both datasets were visualized using the Integrative Genomics Viewer (IGV), and genomic coordinates were based on the hg38 human genome assembly.

### Cell adhesion assay

HASMCs subjected to TCF7L2 overexpression or knockdown were transferred to Opti-MEM for 24 hours before subsequent experiments. Cells were collected, counted, and seeded at equal density onto collagen I–coated 96-well plates. Following a 90-minute incubation at 37°C to allow for cell adhesion, non-adherent cells were removed by washing with PBS. Adherent cells were stained with 0.5% crystal violet, rinsed, and air-dried. Microscopic images were captured, and cell adhesion was quantified by measurement of the crystal violet–positive area using ImageJ software (NIH).

### MMP activity assay

HASMCs were transfected with si*TCF7L2* or control siRNA or infected with Lenti-MMP14 for 24 hours and then maintained in Opti-MEM for an additional 24 hours. Conditioned media were collected and cleared by centrifugation, and total MMP activity was measured using a fluorometric MMP Activity Assay Kit (Abcam, ab112146) according to the manufacturer’s instructions. Fluorescence was recorded on a microplate reader.

### Protein isolation and Western blotting

Cells were lysed on ice for 30 minutes using RIPA lysis buffer (Thermo Fisher Scientific, 89901) supplemented with protease and phosphatase inhibitors. Lysates were then centrifuged at approximately 13,500*g* for 15 minutes at 4°C, and the supernatants were collected and quantified using the Bradford assay. Protein samples were mixed with 4× SDS loading buffer and denatured at 95°C for 10 minutes. Proteins were resolved by SDS-PAGE and transferred to membranes using a Bio-Rad transfer system. Membranes were blocked with 5% nonfat milk in 1× TBST for 1 hour at room temperature, followed by overnight incubation at 4°C with primary antibodies. After 3 washes with TBST, membranes were incubated with secondary antibodies for 1 hour at room temperature and then washed 3 additional times. Protein signals were visualized using a fluorescence imaging system (LI-COR, 9140) and quantified with ImageJ software.

### Luciferase assay

Firefly luciferase reporter construct was custom-designed by cloning of an approximately 100 bp genomic fragment encompassing the ChIP-seq–identified and ChIP-qPCR–validated TCF7L2 binding region near the *MMP14* locus. Plasmid was generated through a vector construction service (VectorBuilder) using the pRP[En]-miniCMV-Luciferase backbone. A7r5 cells were cotransfected with the reporter constructs and a Renilla luciferase control plasmid using Lipofectamine 2000 once cultures approached 70%–80% confluence. After an overnight recovery, cells were treated with Ad*TCF7L2*, Ad*LacZ*, si*TCF7L2*, or siControl. Luciferase activity was measured 48 hours later using a dual-luciferase assay (Promega, E1910), with Renilla luciferase serving as the internal normalization control.

### Gelatin zymography

HASMCs were transfected with 20 nM si*TCF7L2* or siControl at 70% confluence and incubated for 24 hours before switching to serum-free Opti-MEM. After 48 hours, conditioned media were collected, cleared by centrifugation, and mixed with 5× non-reducing loading buffer. Samples were separated by SDS-PAGE in 7.5% polyacrylamide gels containing 0.4% gelatin. After electrophoresis, gels were washed in renaturation buffer, incubated in activation buffer at 37°C for 24 hours, and stained with Coomassie brilliant blue R-250. Gelatin degradation bands were visualized after destaining using an LED transilluminator (57). Band intensities were quantified in ImageJ, using the same procedure as for Western blot analysis.

MMP activities in aortic tissue were assessed using the same method. Briefly, the *Tcf7l2^SMKO^* and floxed control mice were subjected to Ang II–induced AAA, after which proteins were extracted from abdominal aortic tissues. Equal amounts of lysate (20 μg) were separated on 10% SDS-PAGE gels. Gels were incubated in 2.5% Triton X-100 for 60 minutes to remove SDS, then incubated for 24 hours in a developing buffer (50 mmol/L Tris-HCl, 0.2 mol/L NaCl, 5 mmol/L CaCl_2_, and 0.02% Brij-35, pH 7.6). Gel was stained for 1 hour in staining solution containing 0.1% Coomassie blue R-250 (Bio-Rad Laboratories Inc., 1610400), 30% methanol, and 10% glacial acetic acid, and destained for 30 minutes in destaining solution (30% methanol and 10% acetic acid). MMP activity was then visualized as described above.

### Statistics

All statistical analyses were performed with GraphPad Prism 10.0 (general datasets) or R v4.3.3 (RNA-seq, single-cell RNA-seq, and ChIP-seq), while summary data–based Mendelian randomization used SMR v1.3.1 (27). Data are expressed as mean ± SEM. Normality and homoscedasticity were assessed by Shapiro-Wilk and Levene’s tests, respectively; for normally distributed variables, differences between 2 independent groups were analyzed with unpaired 2-tailed Student’s *t* test, whereas non-parametric data were compared with the Mann-Whitney *U* test. Multigroup comparisons used 1-way ANOVA followed by Tukey’s post hoc test or, when normality was violated, the Kruskal-Wallis test with Dunn’s correction; 2-factor experiments were evaluated by 2-way ANOVA with Holm-Šidák multiple comparisons. Categorical variables such as aneurysm incidence were examined with the χ^2^ test, and survival curves were analyzed by the Mantel-Cox log-rank method. Sequencing data were processed in R with DESeq2, Seurat, edgeR, and clusterProfiler, applying Benjamini-Hochberg adjustment for multiple testing. A 2-tailed *P* less than 0.05 was considered statistically significant, and all in vitro experiments were repeated at least 3 times independently, with animal numbers detailed in the corresponding figure legends.

### Study approval

All animal experiments were conducted in accordance with the *Guide for the Care and Use of Laboratory Animals* (National Academies Press, 2011) and were approved by the Institutional Animal Care and Use Committee of the University of Michigan. Human abdominal aortic specimens were obtained from the Cardiovascular Health Improvement Project core of the Frankel Cardiovascular Center at the University of Michigan after written informed consent had been obtained from all participants and with prior approval from the corresponding Institutional Review Board.

### Data availability

The datasets generated and/or analyzed in the current study are available in this publication. Sequencing data are available in the NCBI’s GEO database under accession numbers GSE329070 (RNA-seq) and GSE329072 (ChIP-seq). The AAAGen GWAS summary statistics used in this study are publicly available at https://csg.sph.umich.edu/willer/public/AAAgen2023/

## Author contributions

YD, YL, YZ, XZ, H Liu, ZW, WH, and TZ performed experiments and analyzed the results. YD and JZ wrote the article. GZ, CX, H Lu, YG, LC, and IS provided technical support and contributed to discussions of the project and the article. YD, YEC, and JZ designed the research and discussed the results.

## Conflict of interest

The authors have declared that no conflict of interest exists.

## Funding support

This work is the result of NIH funding, in whole or in part, and is subject to the NIH Public Access Policy. Through acceptance of this federal funding, the NIH has been given the right to make the work publicly available in PubMed Central.

- NIH grants HL134569 (to YEC), HL109946 (to YEC and IS), HL166203 (to YG), HL151524 (to LC), and HL153710 (to JZ).

## Supplementary Material

Supplemental data

Unedited blot and gel images

Supporting data values

## Version 1 04/30/2026

In-Press Preview

## Version 2 06/22/2026

Electronic publication
