# Supplementary material for: TCF7L2 promotes abdominal aortic aneurysm through smooth muscle cell–mediated extracellular matrix remodeling
Source: JCI Insight. 2026 Apr 30;11(12):e195681. doi: 10.1172/jci.insight.195681 (PMC13313545; doi:10.1172/jci.insight.195681)
Supplement: Supplemental data [file jciinsight-11-195681-s038.pdf]

Supplementary materials

for

TCF7L2 Promotes Abdominal Aortic Aneurysm through Smooth Muscle Cell-Mediated Extracellular Matrix Remodeling

Yongjie Deng, Yaozhong Liu, Yang Zhao, Hongyu Liu, Guizhen Zhao, Zhenguo Wang, Xu Zhang, Chao Xue, Wei Huang, Tianqing Zhu, Haocheng Lu, Yanhong Guo, Lin Chang, Ida Surakka, Y. Eugene Chen, Jifeng Zhang

Contents:

Supplemental Figures 1 through 9.

Supplemental Table 1.

Major Resource Table.

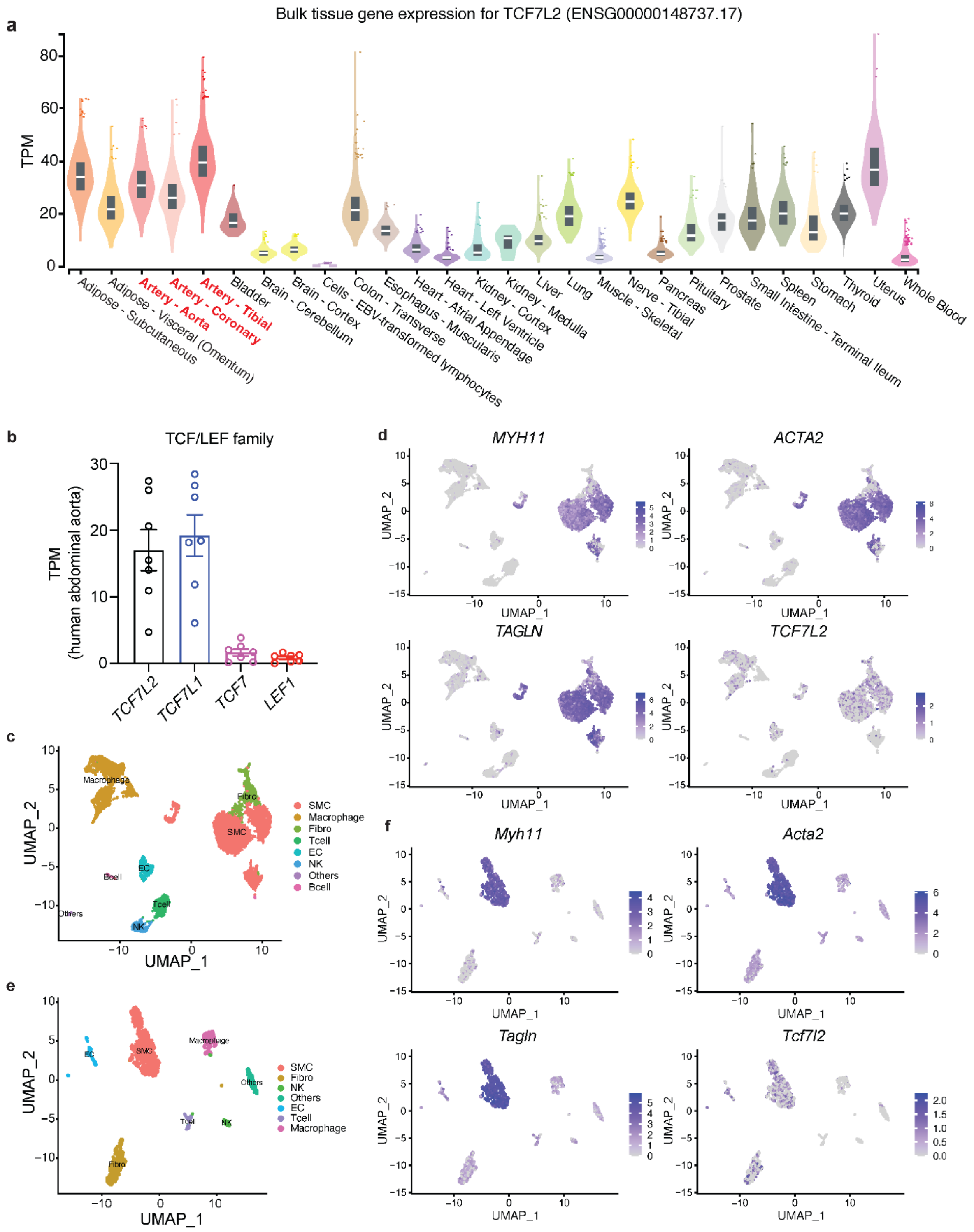

Supplemental Figure 1. *TCF7L2* is enriched in aortic tissue and vascular smooth muscle cells.

(**a**) TCF7L2 expression across multiple human tissues based on the GTEx bulk RNA-seq dataset. (**b**) TPM values of TCF/LEF family members from human abdominal aortic bulk RNA-seq of non-aneurysmal donors. (**c-f**) Clustering and gene expression analysis from

publicly available scRNA-seq datasets. UMAP clustering of human (GSE155468) (**c**) and mouse (GSE152583) aortic cells (**e**). Feature plots of *TCF7L2*, *MYH11*, *ACTA2,* and *TAGLN* expression across human (**d**) and mouse (**f**) aortic cell clusters. Data are presented as mean ± SEM.

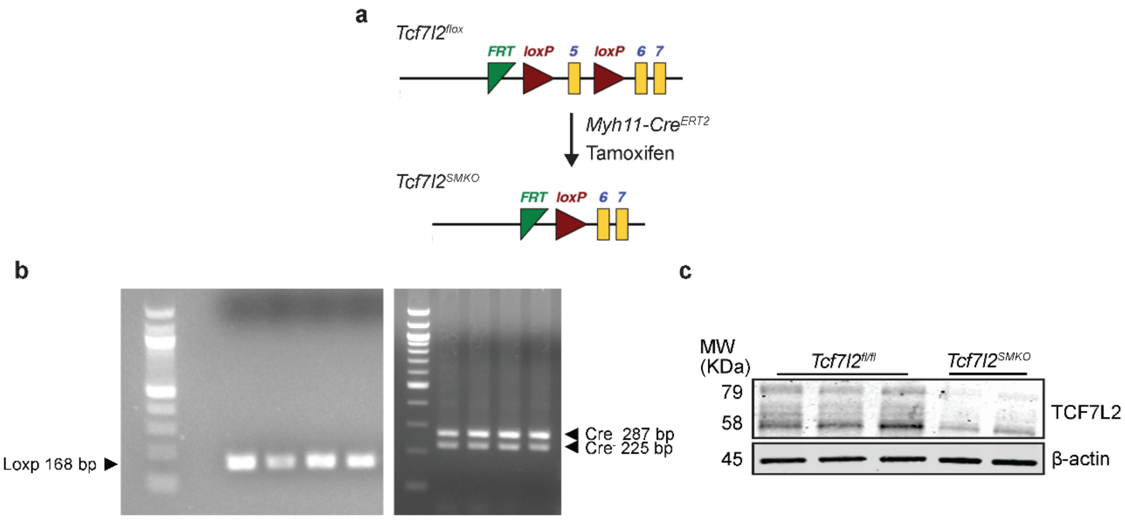

Supplemental Figure 2. Generation and validation of SMC-specific *Tcf7l2* knockout mice.

(**a**) Schematic diagram of the gene targeting strategy to generate smooth muscle cell-specific *Tcf7l2* knockout mice. (**b**) Genotyping of floxed allele and Cre recombinase by standard PCR. (**c**) Protein levels of TCF7L2 and β-actin in abdominal aortic tissues from *Tcf7l2*$^{fl/fl}$ and *Tcf7l2*^{SMKO} mice.

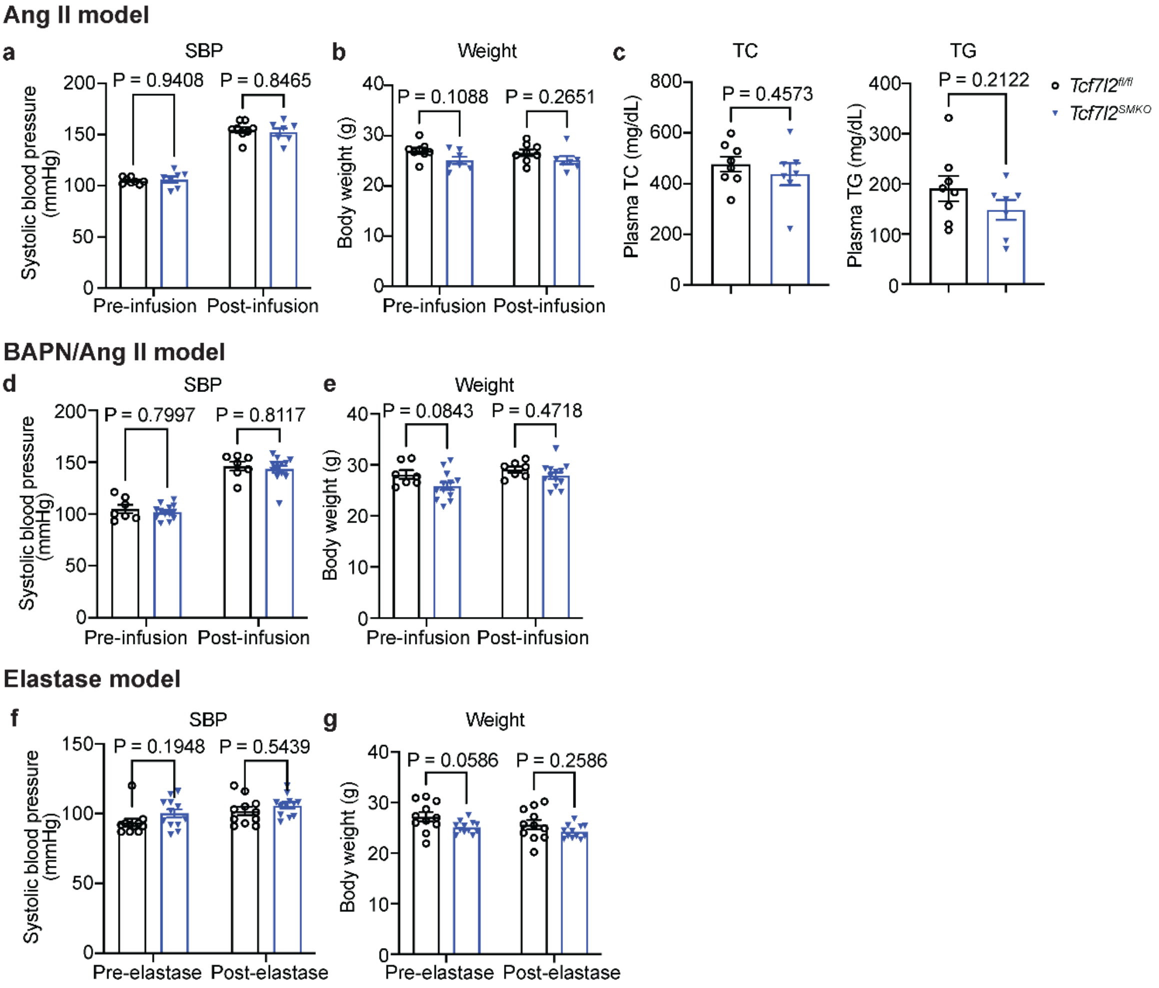

Supplemental Figure 3. SMC-TCF7L2 deficiency does not affect systemic blood pressure, body weight, or lipid levels across three AAA models.

(**a-c**) Systolic blood pressure (**a**), body weight (**b**), serum total cholesterol (TC) and triglyceride (TG) levels (**c**) in *Tcf7l2*$^{fl/fl}$/*Apoe*$^{-/-}$ and *Tcf7l2*^{SMKO}/*Apoe*$^{-/-}$ mice in the Ang II model. (**d-e**) Systolic blood pressure (**d**) and body weight (**e**) in *Tcf7l2*$^{fl/fl}$ and *Tcf7l2*^{SMKO} mice in the BAPN/Ang II model. (**f-g**) Systolic blood pressure (**f**) and body weight (**g**) in *Tcf7l2*$^{fl/fl}$ and *Tcf7l2*^{SMKO} mice in the elastase model. Data are presented as mean ± SEM. P values were calculated using 2-way ANOVA followed by Holm-Šidák post hoc analysis (**a, b, d, e, f, g**) or Student's t test (**c**).

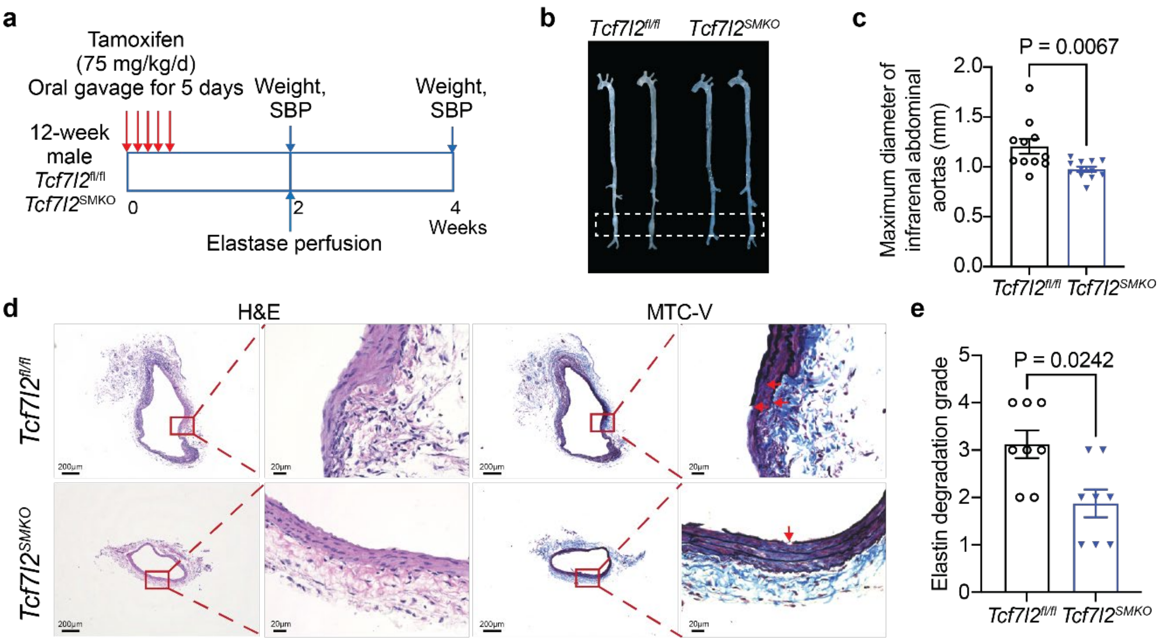

Supplemental Figure 4. SMC-TCF7L2 deficiency alleviates elastase-induced AAA formation.

In the elastase-induced AAA model, 12-week-old male *Tcf7l2$^{fl/fl}$* (n=11) and *Tcf7l2^{SMKO}* (n=12) mice were treated with 30 μL elastase applied to the infrarenal abdominal aortas for 30 minutes. (**a**) Schematic of the elastase-induced AAA model. (**b**) Representative images showing the morphology of aortas at the endpoint. (**c**) Maximum diameter of the infrarenal abdominal aorta. (**d**) Representative images of hematoxylin and eosin (H&E) and Masson's Trichrome-Verhoeff staining (MTC-V) staining of infrarenal abdominal aortas; red arrows indicate elastin degradation. (**e**) Grade of elastin degradation. Data are presented as mean ± SEM. *P* values were calculated using Student's t test (**c**) or Mann-Whitney U test (**e**).

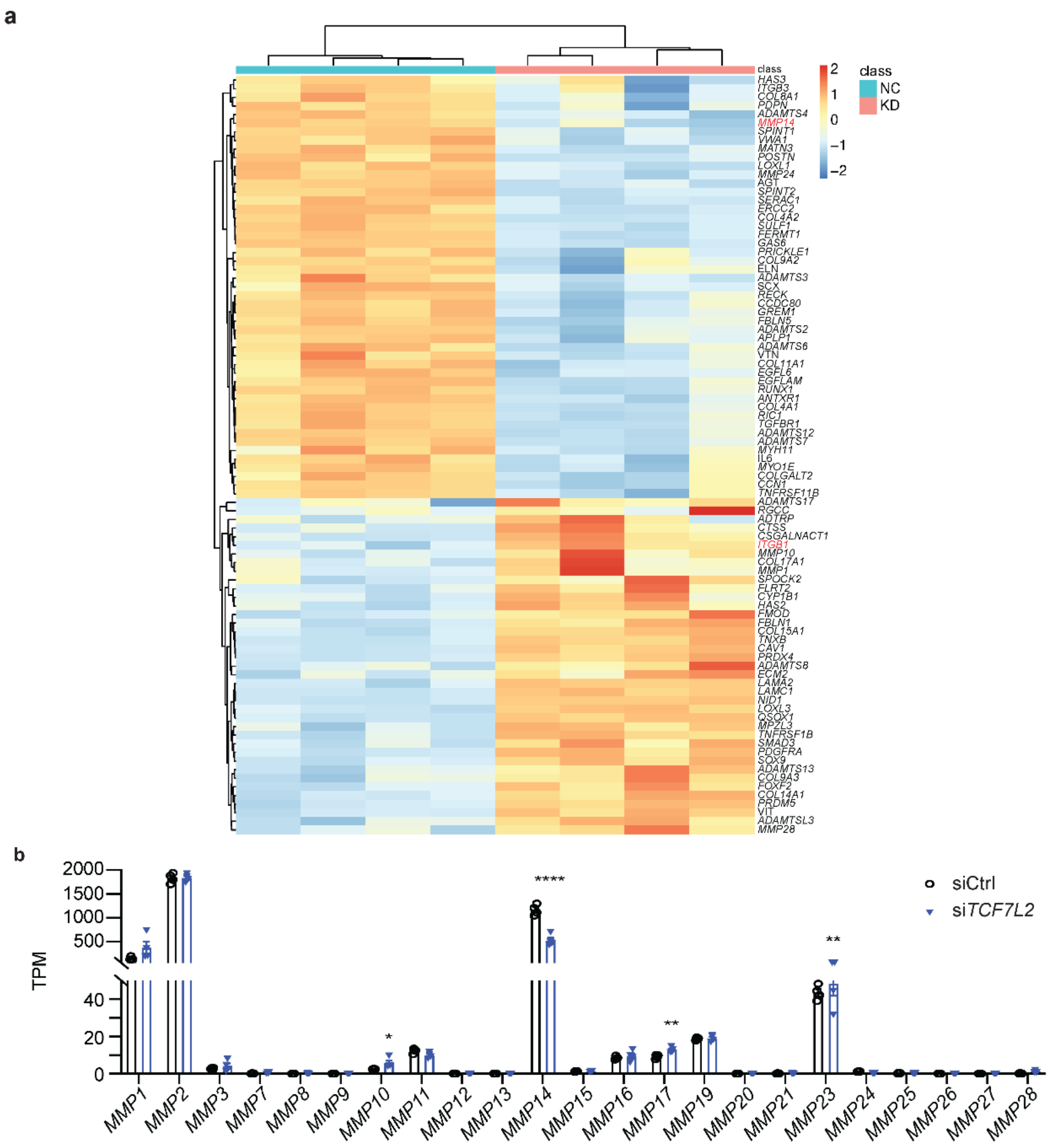

Supplemental Figure 5. Expression profiling of ECM remodeling genes and MMP family members in HASMCs.

(**a**) Heatmap of RNA-seq expression levels of extracellular matrix (ECM) remodeling–associated genes in HASMCs. (**b**) RNA-seq expression levels of matrix metalloproteinase (MMP) family genes in HASMCs. Data are presented as mean ± SEM. *P* values were calculated using Student's t test. TPM = transcripts per million.

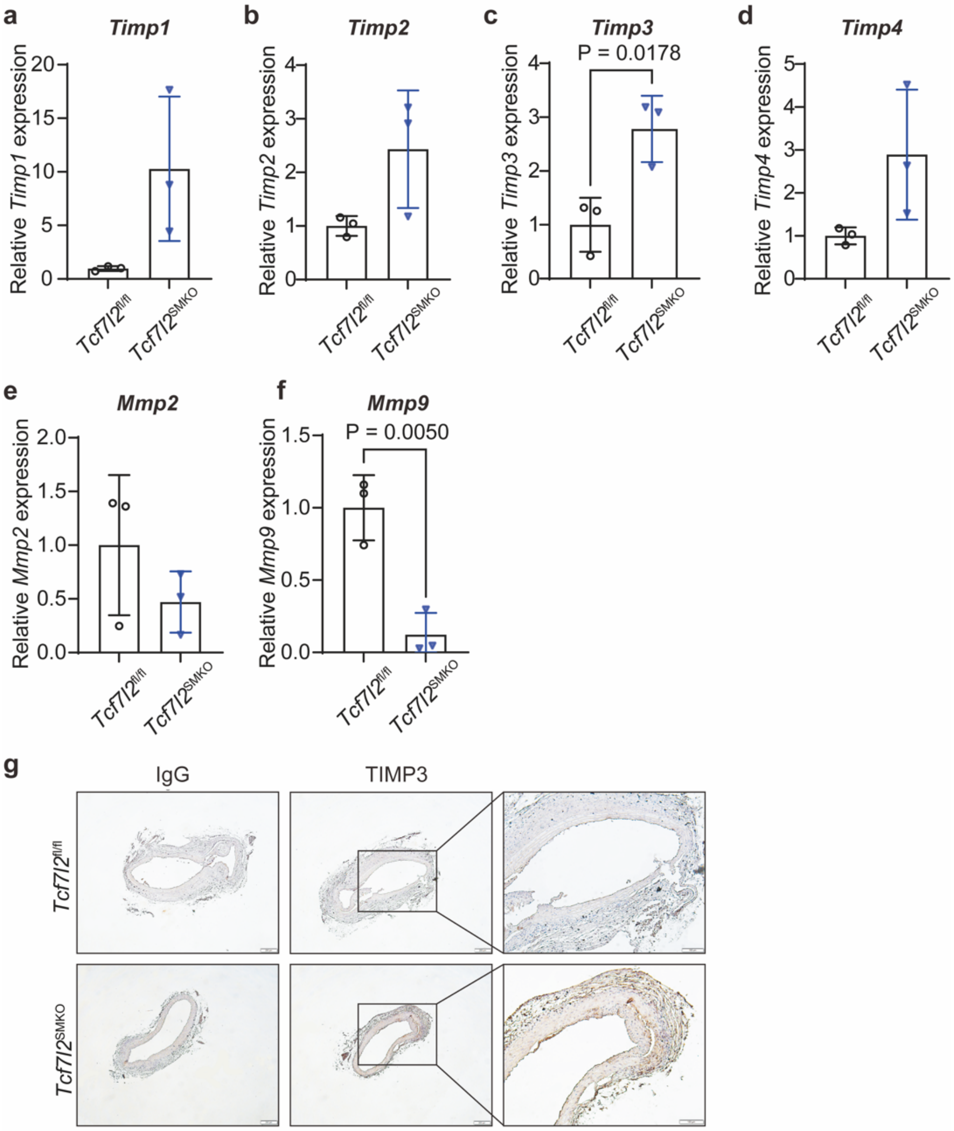

Supplemental Figure 6. *In vivo* validation of TCF7L2-mediated regulation of TIMPs and MMPs in aortic aneurysmal tissues.

(a-f) The *Tcf7l2*^{SMKO} and floxed control mice were subjected to Ang II-induced AAA. After one week, the RNA was isolated from the abdominal aorta. qRT-PCR analysis of mRNA expression levels of tissue inhibitors of metalloproteinases (*Timp1-4*) and *Mmps* in aortic tissues isolated from *Tcf7l2*$^{fl/fl}$ and *Tcf7l2*^{SMKO} mice. Data are presented as mean ± SD (n = 3 per group). (g) Representative immunohistochemical (IHC) staining of TIMP3 in aortic cross-sections from *Tcf7l2*$^{fl/fl}$ and *Tcf7l2*^{SMKO} mice. Isotype-matched IgG was used as a negative control (left panels). Boxed regions are shown at higher magnification in the right panels.

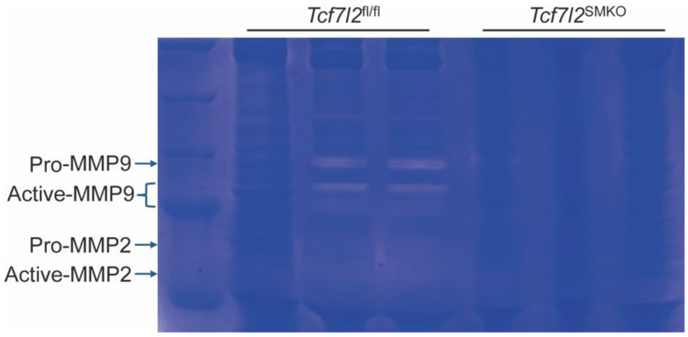

Supplemental Figure 7. Gelatin zymography of abdominal aorta tissue extracts from *Tcf7l2*$^{\mathrm{fl/fl}}$ and *Tcf7l2*^{SMKO} mice.

The *Tcf7l2*^{SMKO} and floxed control mice were subjected to Ang II-induced AAA. After one week, the protein lysate from abdominal aortic tissue was used to assess MMP activity. Representative gelatin zymography showing bands corresponding to the pro- and active forms of MMP9 (upper region) and MMP2 (lower region).

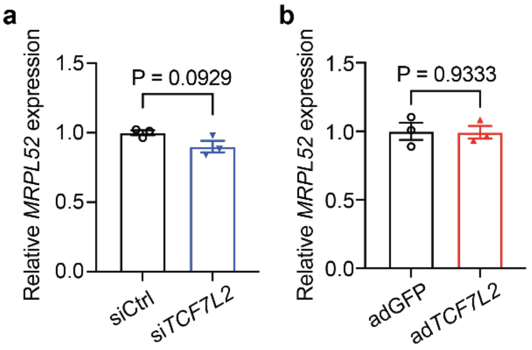

Supplemental Figure 8. TCF7L2 modulation does not alter *MRPL52* expression.

Quantification of *MRPL52* mRNA levels in HASMCs transfected with 20 nM si*TCF7L2* or siControl (**a**), or infected with 20 MOI Ad*TCF7L2* or Ad*GFP* (**b**) for 48 hours, followed by serum starvation in Opti-MEM for 24 hours. Data are presented as mean ± SEM. *P* values were calculated using Student's t test.

a

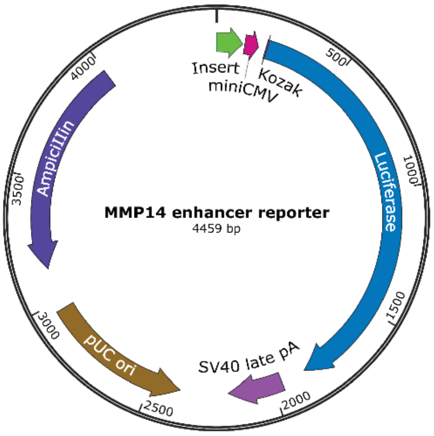

b

Enhancer insert sequence (5'-3'):

TGTTACAGTGATGCGAGGATCTGTAG
AATGACGTGGATGTTACCCCTGAGAT
CAAAGTTACTATTAATATCAATTTCAATG
AACTAGAGACCGCTGTAGGGAGGTTG

Supplemental Figure 9. Map of the MMP14 enhancer luciferase reporter construct.

The plasmid map shows the firefly luciferase reporter construct used in this study, including the mini CMV promoter, luciferase coding sequence, and the inserted enhancer fragment located upstream of MMP14. Vector backbone elements and cloning sites are indicated for reference.

Supplemental Table 1: qPCR primers used in this study.

Primer	Sequences (5'-3')
TCF7L2 (human)	F: GAATCGTCCCAGAGTGATGTC
	R: ACGACCTTTGCTCTCATTTCC
MMP14 (human)	F: GAGCTCAGGGCAGTGGATAG
	R: GGTAGCCCGGTTCTACCTTC
TIMP1 (human)	F: AATTCCGACCTCGTCATCAG
	R: TGCAGTTTTCCAGCAATGAG
TIMP2 (human)	F: AAGCGGTCAGTGAGAAGGAAG
	R: GGGGCCGTGTAGATAAACTCTAT
TIMP3 (human)	F: CTGACAGGTCGCGTCTATGA
	R: GGCGTAGTGTTTGGACTGGT
TIMP4 (human)	F: CCACTCGGCACTTGTGATTC
	R: CATCCTTGACTTTCTCAAACCCT
ITGB1 (human)	F: CAAGAGAGCTGAAGACTATCCCA
	R: TGAAGTCCGAAGTAATCCTCCT
MRPL52 (human)	F: GGACTGTTCTCTTCAGTGTCCG
	R: TCGAAGCTGGCCTTTCATTG
Mmp14 (mouse)	F: CCAAGGCAGCAACTTCAGC
	R: GTGAGCGTTGTGTGTGGGTA
MMP14 (ChIP)	F: GAATGACGTGGATGTTACCCC
	R: GTCCTCCCAGATTAAGCCGT

Major Resource Table

Animals

Species (Mouse)	Source	Stock No.	Sex
Tcf7l2$^{fl/fl}$	International Mouse Phenotyping Consortium (IMPC)	EM:15863	male
Myh11-CreER^{T2}	The Jackson Laboratory	019079	male
Apoe$^{-/-}$	The Jackson Laboratory	002052	male

Cells

Strain	Source	Catalog No.
Human aortic smooth muscle cells (HASMCs)	Lonza	CC-2571
A7r5	ATCC	CRL-1444

Antibodies

Target antigen	Source	Catalog No.	Applications	Working concentration
TCF7L2	Cell Signaling Technology	2569	WB	1 μg/mL
Integrin β1	Cell Signaling Technology	4706	WB	1 μg/mL
β-actin	Cell Signaling Technology	3700	WB	0.5 μg/mL
MMP14	Abcam	ab51074	IF&WB	IF, 5 μg/mL; WB, 1 μg/mL
TIMP3	Proteintech	10858-1-AP	WB	1 μg/mL

IF, immunofluorescence staining; WB, western blot.

Reagents

Reagent	Source	Catalog No.	Working concentration
Angiotensin II	Bachem	4006473	1000 ng/kg/min
BAPN	Sigma-Aldrich	A3134	150 mg/kg/day
Elastase	Sigma-Aldrich	E1250	44 units/mL
si*TCF7L2*	Horizon Discovery	D-003816-01	20 nM
si*ITGB1*	Horizon Discovery	L-004506-00	20 nM
siCtrl	Horizon Discovery	D-001206-14	20 nM

Kits

Reagent	Source	Catalog No.
Cholesterol E	Fujifilm Wako	999-02601
L-type Triglyceride M	Fujifilm Wako	632-50991
IHC kit	Abcam	Ab64261
ChIP assay Kit	Cell Signaling Technology	9003
MMP activity assay kit	Abcam	Ab112146
Dual-luciferase assay kit	Promega	E1910
