# Supplementary material for: TCF7L2 promotes abdominal aortic aneurysm through smooth muscle cell–mediated extracellular matrix remodeling
Source: JCI Insight. 2026 Apr 30;11(12):e195681. doi: 10.1172/jci.insight.195681 (PMC13313545; doi:10.1172/jci.insight.195681)

Full unedited blot for Figure 4e

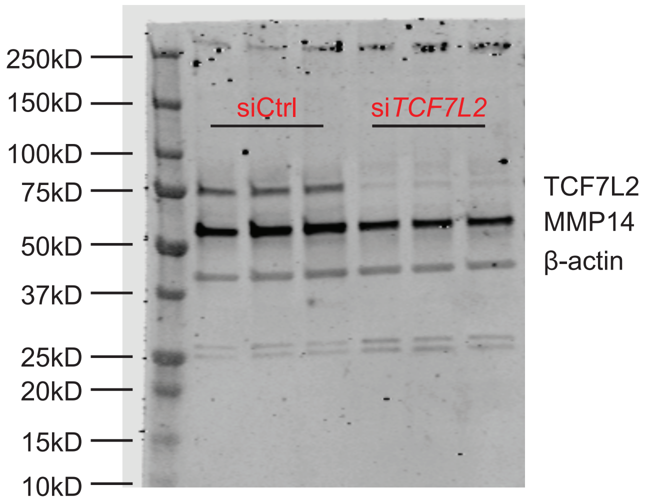

Full unedited blot for Figure 4g

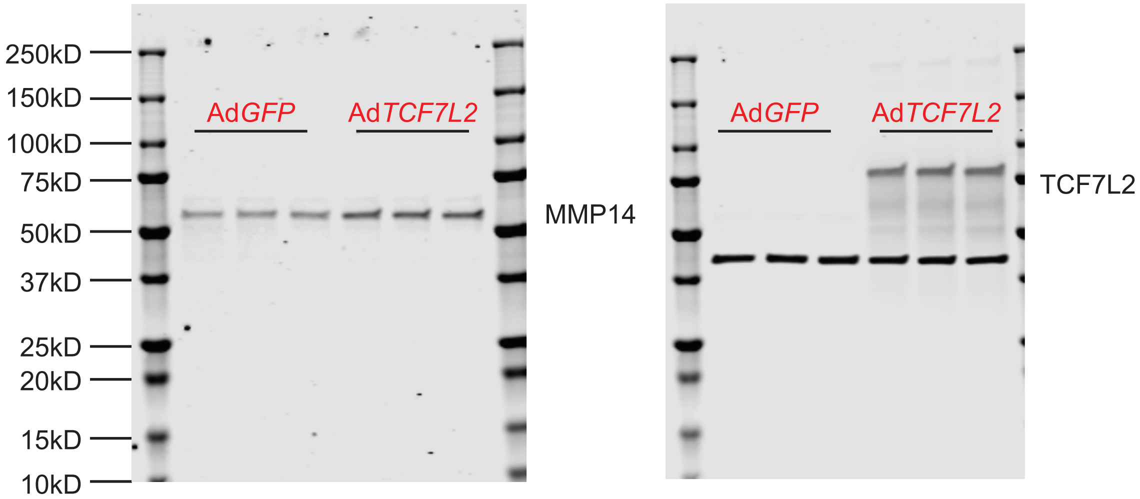

Full unedited blot for Figure 5d

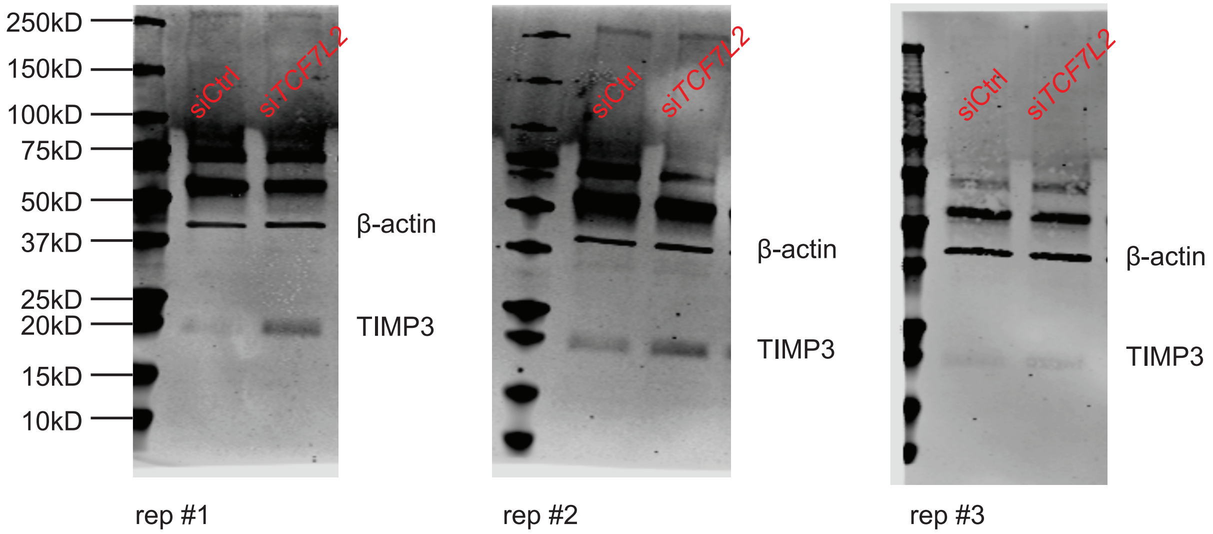

Full unedited blot for Figure 5f

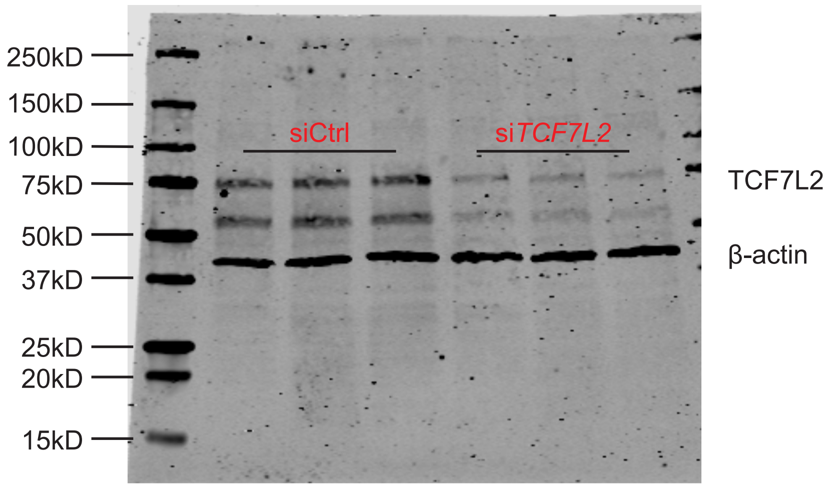

Full unedited blot for Figure 6f

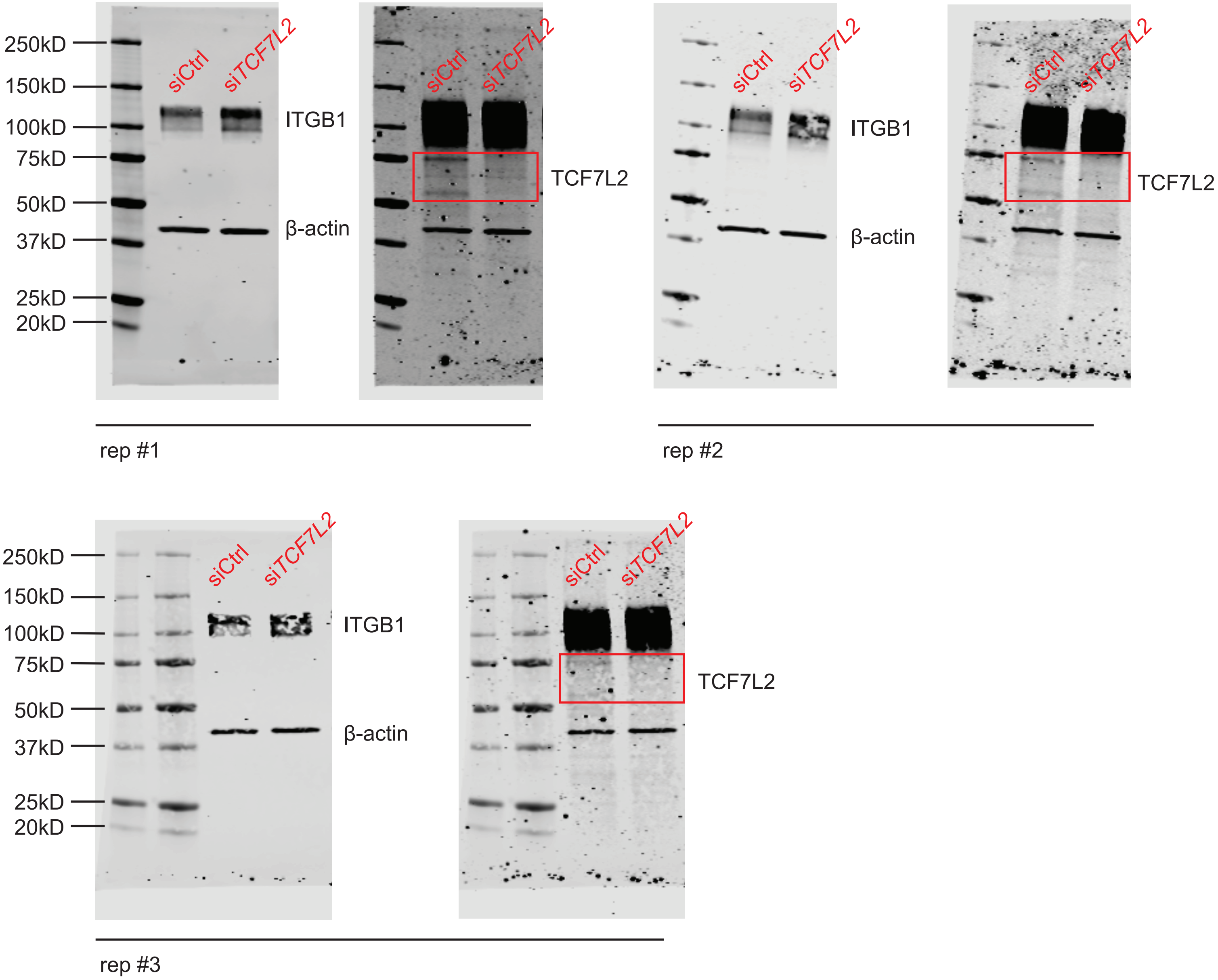

Full unedited blot for Figure 6i

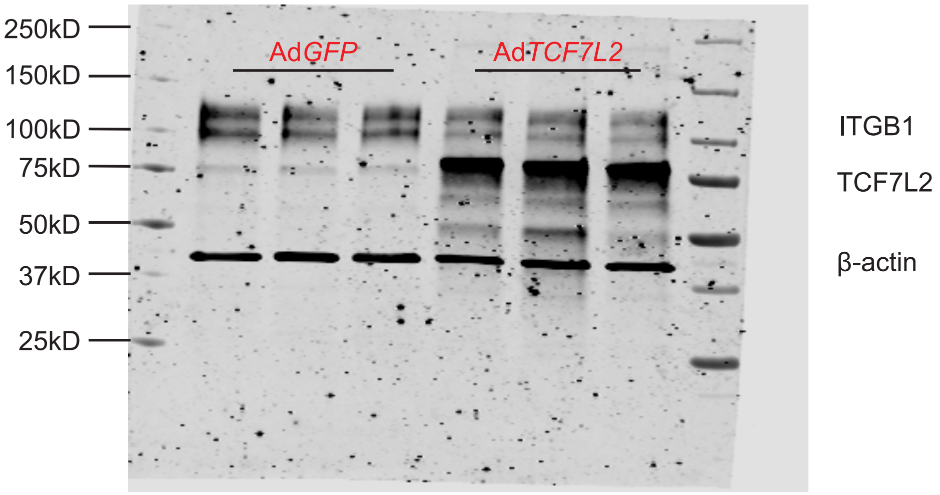

Full unedited blot for Supplemental Figure 2c

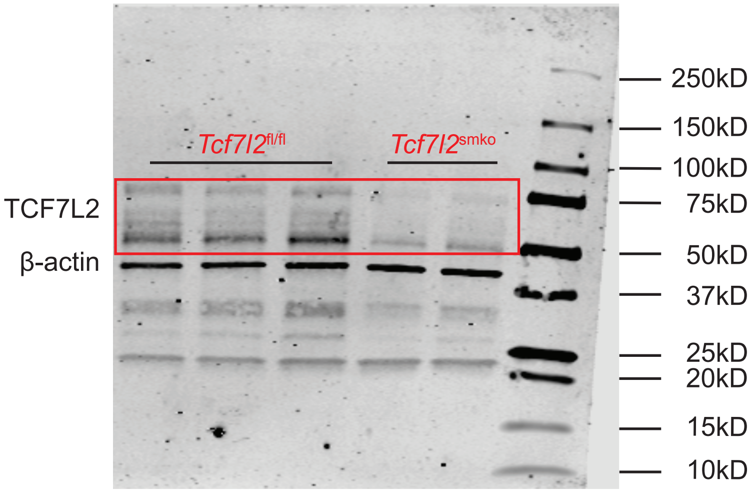

Supplement: Unedited blot and gel images [file jciinsight-11-195681-s039.pdf]
